# An Ion‐Based Strategy Toward Synergistic Surface Functionalization Combining the Osteogenic Properties and NIR‐Mediated Antibacterial Activity of PEEK

**DOI:** 10.1002/adma.73945

**Published:** 2026-07-09

**Authors:** Zhiyan Xu, Ondřej Adam, Marek Doubrava, Ana M. Beltrán, Ivo Dlouhý, Xin Liu, Baiyan Sui, Aldo R. Boccaccini

**Affiliations:** ^1^ Institute of Biomaterials Department of Materials Science and Engineering University of Erlangen‐Nuremberg Erlangen Germany; ^2^ Institute of Materials Science and Engineering Technicka 2 Brno University of Technology Brno Czech Republic; ^3^ Departamento de Ingeniería y Ciencia de los Materiales y del Transporte Escuela Politécnica Superior Universidad de Sevilla Sevilla Spain; ^4^ Institute of Physics of Materials Czech Academy of Sciences Brno Czech Republic; ^5^ Department of Dental Materials Shanghai Biomaterials Research & Testing Center College of Stomatology National Center for Stomatology National Clinical Research Center for Oral Diseases Shanghai Ninth People's Hospital Shanghai Jiao Tong University School of Medicine Shanghai Jiao Tong University Shanghai Key Laboratory of Stomatology Shanghai P. R. China

**Keywords:** DFT calculation, ion‐assisted therapy, NIR‐mediated phototherapy, PEEK, surface functionalization

## Abstract

Persistent implant‐associated infections critically compromise the longevity and success of orthopedic prostheses. Herein, we present a facile surface‐engineering strategy to construct a multifunctional PEEK‐based implant by introducing manganese‐chelated polydopamine (Mn@p) and mesoporous bioactive glass nanoparticles (MBGNs) onto sulfonated PEEK (SPEEK). The resulting Mn@p/MBG‐SPEEK exhibits a hierarchically porous three‐dimensional architecture with markedly enhanced hydrophilicity and surface roughness. This tailored interface shows in vitro bioactivity and cytocompatibility, significantly promoting the osteogenic differentiation of MC3T3‐E1 cells through the activation of PI3K/Akt/mTOR and AP‐1 signaling pathways, as evidenced by the upregulation of ALP, OCN, OPN, and Runx2 expression. Meanwhile, the pro‐angiogenic potential of Mn@p/MBG‐SPEEK is supported by the upregulation of VEGF and CD31 in HUVECs and enhanced capillary‐like network formation. Notably, Mn@p/MBG‐SPEEK demonstrates efficient light‐to‐heat conversion and potent antibacterial efficacy against *Staphylococcus aureus* under near‐infrared (NIR) irradiation. Density functional theory (DFT) calculations further reveal that Mn chelation narrows the HOMO‐LUMO energy gap and facilitates charge separation, thereby amplifying photothermal and ROS‐mediated antibacterial effects. Collectively, this study establishes a versatile and scalable route to enhance the biological performance of PEEK implants, offering a conceptual framework for integrating ion‐assisted therapy with phototherapy toward next‐generation bioactive and infection‐resistant orthopedic materials.

## Graphical abstract

A multifunctional bioactive PEEK‐based implant (Mn@p/MBG‐SPEEK) is engineered to combat implant‐associated infections while promoting osteogenesis. The hybrid surface activates PI3K/Akt/mTOR and AP‐1 signaling pathways for osteogenic differentiation, while Mn coordination induces electronic coupling that narrows the HOMO‐LUMO energy gap and enhances charge separation, enabling efficient photothermal and ROS‐driven antibacterial effects.

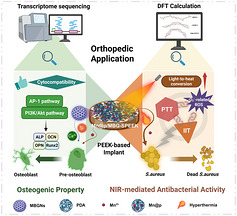

## 1 Introduction

Polyetheretherketone (PEEK) has emerged as a prospective alternative to traditional metallic implants in orthopedic applications due to its favorable biocompatibility, suitable mechanical performance, high thermal stability, non‐toxicity, tunability for molding and processing, chemical inertness, and radiolucency [1, 2, 3, 4]. In addition, its elastic modulus (2–6 GPa) is close to that of human trabecular bone, thereby effectively minimizing stress shielding effects [1, 5, 6]. Despite these advantages, PEEK remains hydrophobic and bioinert, resulting in limited osteointegration and osteogenesis [6]. Consequently, such limitations can increase the risk of implant‐related infections (IRIs), one of the most critical complications in orthopedic surgery, leading to prolonged inflammation, implant failure, increased healthcare costs, and severe morbidity [7, 8, 9, 10, 11]. Moreover, the rising incidence of antibiotic‐resistant bacterial strains and biofilm formation further intensifies these challenges, highlighting the urgent need to develop PEEK‐based orthopedic implants with robust antimicrobial properties and enhanced osteogenic performance.

As a non‐invasive therapeutic strategy, near‐infrared (NIR)‐mediated photothermal therapy (PTT) has attracted widespread attention in the field of exogenous antibacterial treatment [11], owing to its broad‐spectrum antimicrobial activity, effectiveness against antibiotic‐resistant bacteria and biofilms, rapid treatment course, and minimal risk of inducing bacterial resistance [12, 13]. Among the diverse available photothermal agents (e.g., transition metal nanomaterials, carbon‐based nanomaterials, and conjugated functional polymers), melanin‐like polydopamine (PDA) has garnered considerable attention due to its easy and cost‐effective production strategy, excellent biocompatibility, hydrophilicity, and redox activity [14, 15]. Moreover, through the self‐polymerization of dopamine, PDA can be easily formed as a thin nanolayer on various substrates via covalent and non‐covalent bonding, and the process is virtually geometry‐ and material‐independent [16]. This feature allows the beneficial performance of PDA to be imparted to various material surfaces. However, PDA alone provides transient antibacterial effects and fails to intrinsically stimulate osteogenesis.

To overcome these limitations, ion‐induced therapy (IIT), which involves bioactive metallic ions, has emerged as a promising approach in the field of regenerative therapies [17]. Building upon this capability, PDA has shown a strong affinity to coordinate/chelate metal ions (e.g., Cu^2+^, Co^2+^, Mn^2+^, Ca^2+^, Mg^2+^, Gd^3+^, and Li^+^) via catechol groups to form metal‐oxygen coordination bonds [18], which has sparked inspiration for combining IIT with PTT to enhance the therapeutic efficacy of PEEK prosthesis. Among various ions, Mn^2+^ has received particular attention due to its significant antibacterial effects, capacity to regulate bone metabolism, and potential to promote osteogenic differentiation [19, 20, 21]. As a transition metal ion, Mn^2+^ may also participate in redox reactions and Fenton‐like processes, thereby contributing to ROS‐involved antibacterial activity under appropriate microenvironmental conditions [22, 23]. Meanwhile, bioactive glass (BG) is another promising candidate material for further enhancing the bioactivity of PEEK surfaces, as it can form bone‐like apatite on the surface when in contact with body fluids and bond to bone in vivo [24]. In particular, mesoporous bioactive glass nanoparticles (MBGNs) with unique texture characteristics can further enhance osteogenic bioactivity by releasing soluble Si‐containing species and Ca^2+^ ions that stimulate biomineralization and cell differentiation [25]. Nevertheless, the synergistic interplay among Mn‐ion‐mediated IIT, PDA‐assisted PTT activity, MBGNs‐induced bioactivity, and vascularization‐supportive responses remains insufficiently explored, representing an untappe opportunity to boost the clinical performance of PEEK‐based orthopedic implants.

It is worth noting that a comprehensive understanding of the fundamental mechanisms underlying photothermal energy conversion and NIR‐induced antibacterial activity of this system at the molecular and electronic levels remains limited yet critically crucial for optimizing the therapeutic design of PEEK implants. Density functional theory (DFT) and time‐dependent DFT (TD‐DFT) theoretical calculations, based on quantum mechanics, can provide a powerful tool for analyzing structural energetics, electronic properties, and excited‐state behaviors, thereby elucidating the potential mechanisms and optimizing the therapeutic outcomes of biofunctional PEEK implants [26].

**FIGURE 1:**
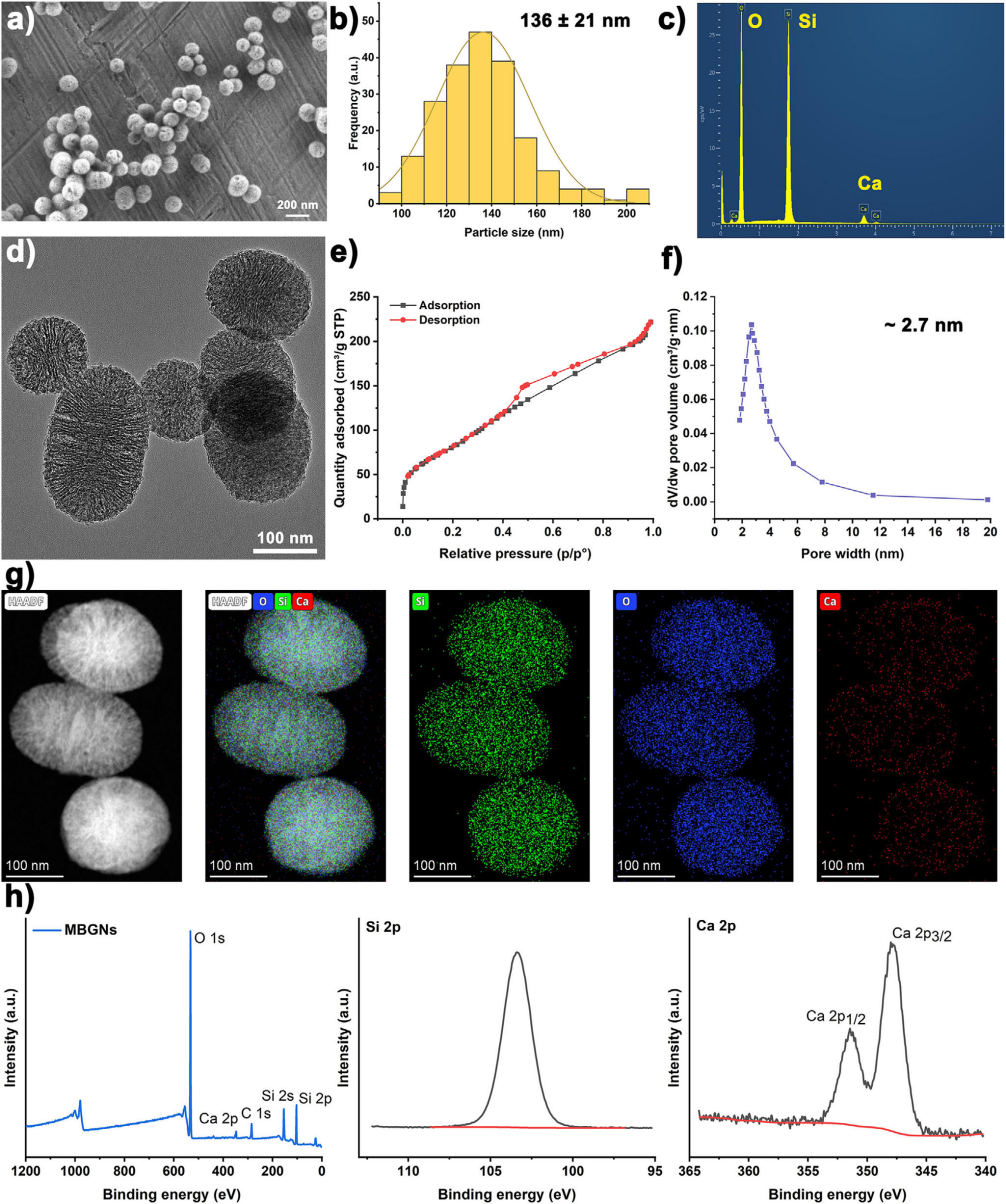
Characterization of synthesized MBGNs, including (a) SEM image, (b) particle size distribution by statistical analysis from SEM images, (c) EDS spectrum, (d) TEM observation, (e) N_2_ adsorption‐desorption isotherm, (f) pore size distribution, (g) STEM‐HAADF image and elemental mapping analyses, (h) XPS survey spectrum and high‐resolution spectra of Si 2p and Ca 2p.

**FIGURE 2:**
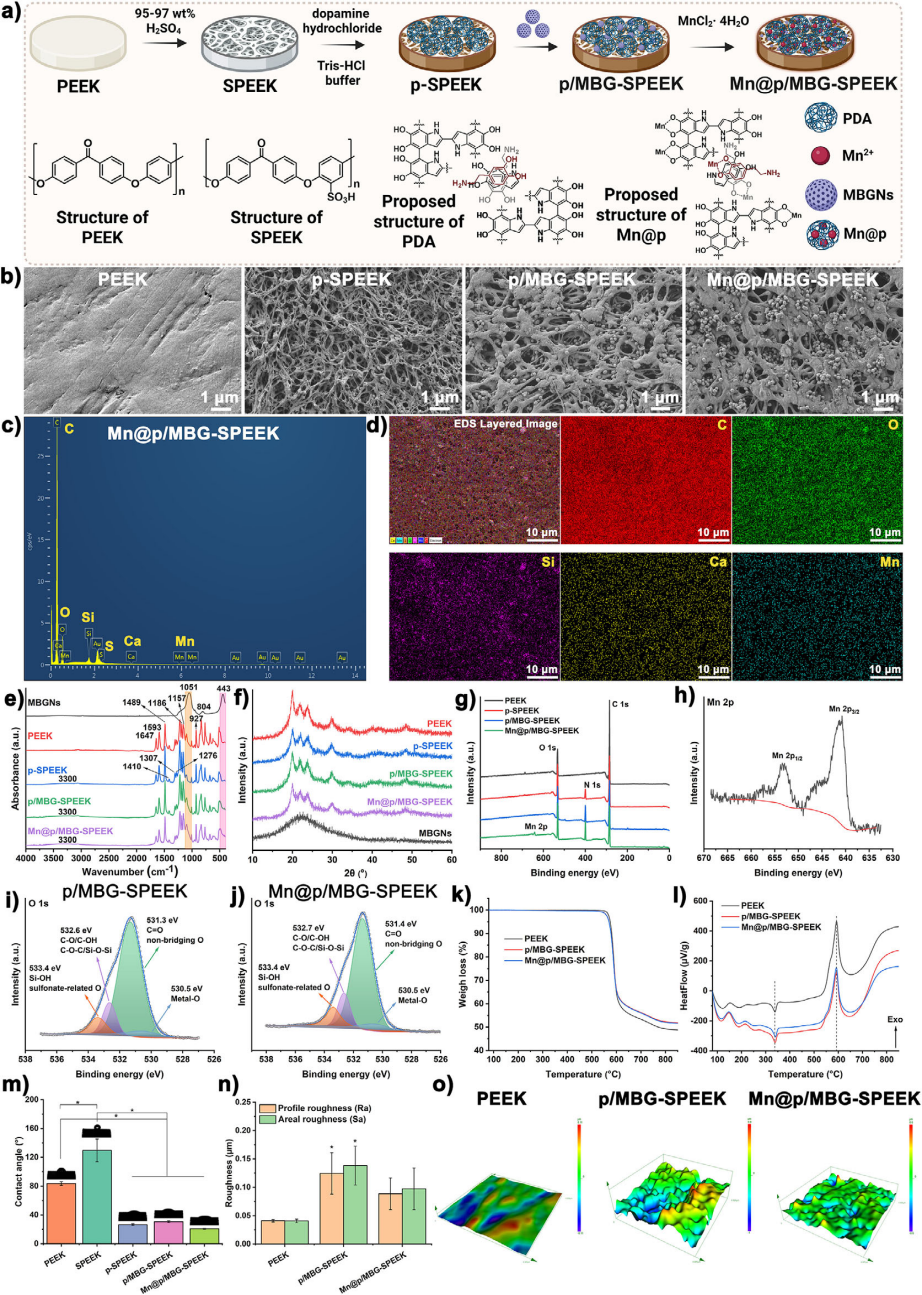
Material design illustration and characterizations of PEEK‐based samples. (a) Schematic illustration: Fabrication route of the multifunctional Mn@p/MBG‐SPEEK implant surface and corresponding chemical structures of PEEK, SPEEK, proposed PDA, and the proposed Mn‐chelated PDA. (b) SEM images of different PEEK samples. (c) EDS spectrum summed from elemental mapping; (d) SEM‐EDS layered image and EDS elemental mapping analyses of Mn@p/MBG‐SPEEK. (e) FTIR spectra and (f) XRD patterns of MBGNs and PEEK‐based samples. (g) XPS survey spectra of PEEK, p‐SPEEK, p/MBG‐SPEEK, and Mn@p/MBG‐SPEEK. (h) High‐resolution Mn 2p spectrum of Mn@p/MBG‐SPEEK. (i,j) High‐resolution O 1s spectra of (i) p/MBG‐SPEEK and (j) Mn@p/MBG‐SPEEK. (k) TGA mass loss and (l) DSC curves of PEEK, p/MBG‐SPEEK, and Mn@p/MBG‐SPEEK. (m) Water contact angle assay of different PEEK samples (*n* = 3); (n) Surface roughness and (o) representative 3D laser scanning images of PEEK, p/MBG‐SPEEK, and Mn@p/MBG‐SPEEK (*n* = 3). Data are presented as mean ± SD. Statistical significance was analyzed by one‐way ANOVA followed by Tukey's HSD post hoc test. ^*^ *p* < 0.05.

Herein, we propose an ion‐based surface modification strategy to improve the osteogenic, angiogenic, and antibacterial activity of PEEK implants. First, we used a microemulsion‐based sol–gel method to synthesize SiO_2_‐CaO MBGNs (Figure 1). Through a facile impregnation approach, PDA and MBGNs were gradually deposited on the surface of sulfonated PEEK (SPEEK), resulting in p‐SPEEK and p/MBG‐SPEEK. Subsequently, Mn ions were incorporated onto p/MBG‐SPEEK by the chelation process. A schematic illustration of the chemical structures and fabrication process of Mn@p/MBG‐SPEEK is presented in Figure 2a. The obtained Mn@p/MBG‐SPEEK was presumed to exert enhanced bioactivity, osteogenesis, angiogenesis, and antibacterial activity. The physicochemical properties, hydrophilicity, surface roughness, in vitro bioactivity, cytocompatibility, osteogenic property, angiogenic potential, photothermal conversion performance, and antibacterial efficacy without/with NIR irradiation of the fabricated materials were thoroughly investigated. Moreover, transcriptome sequencing analysis was applied to demonstrate the activation of osteogenic‐associated signaling pathways. Significantly, we report the development of a Mn‐incorporated hybrid molecular system based on SPEEK, and its photoexcitation behavior was elucidated through DFT and TD‐DFT simulations. Moreover, the experimental evaluations and DFT/TD‐DFT simulations were combined to understand the NIR‐triggered antibacterial mechanism, revealing that Mn coordination enhanced visible‐to‐NIR absorption, narrowed the optical/electronic gap, promoted electron–hole separation, and supported ROS‐involved antibacterial activity, thereby establishing a mechanistically guided strategy for multifunctional implant disinfection and vascularized bone regeneration.

## 2 Results and Discussion

### 2.1 Characterization of MBGNs

Binary SiO_2_‐CaO MBGNs were synthesized employing a microemulsion‐assisted sol–gel method under basic catalytic conditions, referred to as the modified Stöber method [27]. FE‐SEM (Figure 1a) revealed predominantly uniform spherical particles with occasional ellipsoids and distinct surface nanopores, while TEM (Figure 1d) showed well‐organized worm‐like and lamellar mesoporous channels. The particle diameters ranged from 100 to 200 nm, averaging 136 ± 21 nm (Figure 1b). Nitrogen (N_2_) adsorption‐desorption isotherms (Figure 1e) exhibited a type IV profile with a hysteresis loop characteristic of mesoporous structures [28], and the BJH pore size distribution indicated uniform pores centered at around 2.7 nm (Figure 1f). The MBGNs possessed a high BET specific surface area (SSA, 326 ± 4 m^2^ g^−1^) and cumulative pore volume (0.36 cm^3^ g^−1^), as listed in Table S1, conferring abundant active sites for ion exchange, biomineralization, and post‐functionalization. EDS spectra during SEM observation and elemental mappings during STEM‐HAADF imaging (Figure 1c,g) confirmed a homogeneous Si─Ca─O composition and elemental distribution of the MBGNs. The semi‐quantitative EDS analysis indicated a composition of approximately 90.4 mol% SiO_2_ and 9.6 mol% CaO (Table S1), which was further verified by ICP‐OES analysis (Table S1), yielding 91.1 mol% SiO_2_ and 8.9 mol% CaO. XPS analysis, shown in Figure 1h, including the survey spectrum and high‐resolution Si 2p and Ca 2p spectra, further confirmed the presence of Si, O, and Ca on the surface of MBGNs. Furthermore, FTIR spectra (Figure 2e) displayed typical Si‐O‐Si stretching, wagging, and rocking vibrations at 1051, 804, and 443 cm^−1^, respectively [29, 30]. The broad halo observed in the XRD pattern (Figure 2f) in the 15°–35° 2θ range further verified the amorphous nature of the MBGNs. Collectively, these physicochemical features, including a uniform mesoporous morphology, a high specific surface area, and a stable amorphous SiO_2_‐CaO framework, provide a favorable structural and compositional foundation for subsequent ion release, bioactivity, and surface functionalization on PEEK substrates.

### 2.2 Characterizations of PEEK‐Based Samples

Surface topography and chemistry represent key design parameters for optimizing the biological response at the implant‐tissue interface [31]. In Figure 2b, the pristine PEEK surface exhibited a relatively smooth texture, whereas the p‐SPEEK developed a three‐dimensional (3D) porous network introduced by the sulfonation process. Micron‐scale voids (∼3 µm) interspersed with submicron pores (<1 µm) formed a hierarchical micro‐ and nano‐architecture that enhances bone bonding and regeneration [32, 33]. The PDA coating maintained the overall morphology of SPEEK. After MBGNs deposition, the nanoparticles were uniformly distributed along the network filaments and partially embedded within pores, resulting in a well‐defined 3D loading architecture. Following Mn^2+^ chelation, the obtained Mn@p/MBG‐SPEEK retained the porous architecture with homogeneously dispersed nanoparticles, while occasional nanoscale clusters were observed, possibly related to localized Mn‐chelated PDA (Mn@PDA) aggregation. Moreover, elemental analysis confirmed the incorporation of MBGNs and Mn^2+^. The EDS spectrum (Figure 2c) showed peaks for Si, Ca, and Mn, while the elemental mappings (Figure 2d) revealed uniform distributions of Ca and Mn, indicating homogeneous functionalization. Additionally, the S element was also observed (Figure 2c) due to the sulfonic species introduced by sulfuric acid treatment.

The surface chemical composition and crystal structure were further characterized by FTIR and XRD analyses. The FTIR spectra (Figure 2e) exhibited characteristic bands of PEEK in all PEEK‐based samples [34, 35, 36], while MBGNs‐loaded samples displayed intensified Si─O─Si features (Figure S1a), consistent with the presence of MBGNs. Despite the overlap of MBGNs and PEEK characteristic bands, the band intensities varied between the MBGNs‐loaded and pristine PEEK samples. As shown in the zoomed FTIR spectra (1400–400 cm^−1^), p/MBG‐SPEEK and Mn@p/MBG‐SPEEK exhibited increased band intensities at 1109, 1095, 1051, and 443 cm^−1^ (Figure S1a). A new band at 1028 cm^−1^ (Figure S1a), attributed to S═O stretching, verified the successful introduction of sulfonic groups [37, 38], and a broad band near 3300 cm^−1^ (Figure S1b) corresponded to O─H or N─H stretching from PDA formation [35]. The XRD patterns (Figure 2f) showed comparable PEEK crystalline profiles across all samples, indicating that surface modification did not alter the semi‐crystalline structure of the substrate. XPS analysis further confirmed the surface chemical composition of different PEEK samples (Figure 2g,h; Figure S2). High‐resolution Mn 2p and O 1s spectra supported that Mn was incorporated onto Mn@p/MBG‐SPEEK through Mn─O coordination with PDA (Figure 2i,j). Mn incorporation modestly increased the metal‐O‐related component at around 530.5 eV and induced slight shifts in the C─O/C─OH─and C=O‐related components.

Thermal properties remained unchanged, as evidenced by TGA (Figure 2k) and DSC (Figure 2l) curves that overlapped closely among PEEK, p/MBG‐SPEEK, and Mn@p/MBG‐SPEEK. Furthermore, p/MBG‐SPEEK and Mn@p/MBG‐SPEEK exhibited lower mass losses of 48% compared to a 51% loss for PEEK, highlighting the presence of MBGNs. These findings confirmed that the modifications were confined to the surface layer. Surface hydrophilicity and roughness values further indicated the successful modulation of the surface physicochemical characteristics. The water contact angle (Figure 2m) decreased from pristine PEEK to Mn@p/MBG‐SPEEK, indicating enhanced hydrophilicity. The roughness parameters (Figure 2n), including profile roughness (Ra) and areal roughness (Sa), and 3D laser‐scanning images (Figure 2o) of PEEK‐based sample surfaces revealed increased micro‐ and nano‐features after the modifications. Together, these results demonstrate the successful stepwise surface modification of Mn@p/MBG‐SPEEK, in which hierarchical porosity, chemical functionalization, and nanoparticle integration generate a potentially bioactive and structurally stable surface. This well‐defined surface enables subsequent investigations on apatite formation, osteogenic cellular activity, and antibacterial performance.

### 2.3 In Vitro Apatite‐Forming Bioactivity

**FIGURE 3:**
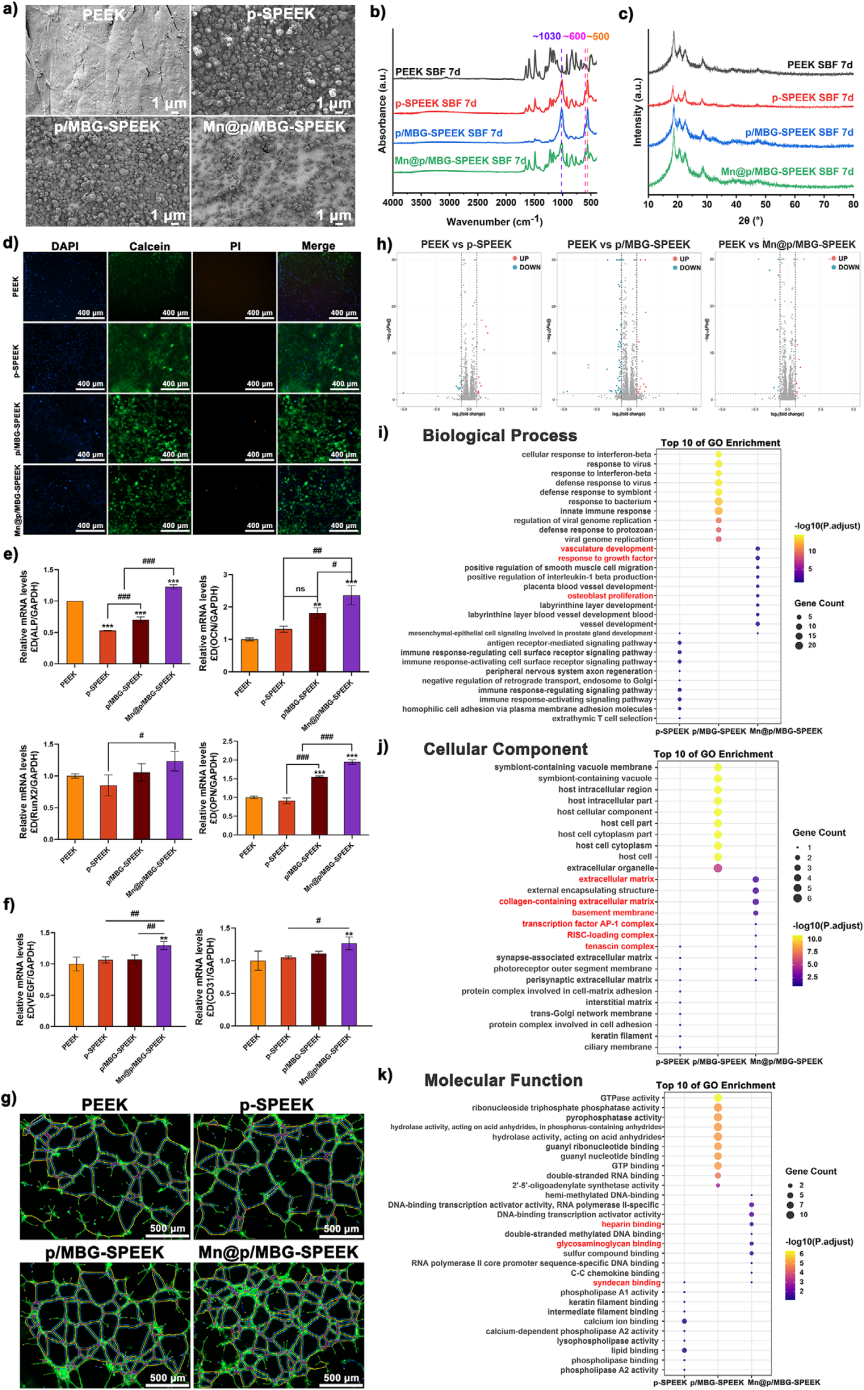
In vitro assessments of bioactivity, cytocompatibility, angiogenic and osteogenic potential of PEEK samples. (a) SEM images, (b) FTIR spectra, and (c) XRD patterns of different PEEK surfaces after immersion in SBF for 7 days. (d) Representative fluorescent images of live/dead stained MC3T3‐E1 cells co‐cultured with PEEK samples for 3 days. (e) Real‐time PCR quantitative analysis of osteogenesis‐associated genes expression of ALP, Runx2, OCN, and OPN in MC3T3‐E1 co‐culture with PEEK samples for 3 days (*n* = 3). (f) Real‐time PCR quantitative analysis of angiogenesis‐associated gene expression of VEGF and CD31 in HUVECs cultured on various PEEK samples for 3 days (*n* = 3). (g) Representative fluorescent images and skeletonized network analysis of tube formation by HUVECs‐GFP treated with extracts from various PEEK samples for 6 h. (h–k) Transcriptomic profiling and GO analysis of MC3T3‐E1 cells cultured on different PEEK‐based substrates: (h) Volcano plots showing differentially expressed genes (DEGs) for each modified group (p‐SPEEK, p/MBG‐SPEEK, Mn@p/MBG‐SPEEK) compared with PEEK. Red dots indicate upregulated DEGs, blue dots indicate downregulated DEGs, and gray dots indicate non‐DEGs; (i–k) GO enrichment analysis of upregulated DEGs for each modified group vs. PEEK, showing the top enriched terms in i) BP, (j) CC, and (k) MF categories. Data are presented as mean ± SD. Statistical significance was evaluated by one‐way ANOVA followed by Tukey's HSD post hoc test. ^*^ *p* < 0.05, ^**^ *p* < 0.01, and ^***^ *p* < 0.001 vs. PEEK; ns: *p* > 0.05, ^#^ *p* < 0.05, ^##^ *p* < 0.01, and ^###^ *p* < 0.001 between indicated groups.

The bioactivity of implantable biomaterials refers to their ability to establish a robust mechanical bond with living tissue, which can be assessed in vitro through immersion in SBF to mimic implantation in the human body [39]. The formation of a bone‐like apatite layer, considered as a biomimetic interface, on the biomaterial surface after immersion in SBF has been proposed to potentially possess bone‐bonding capability [40]. After 7 days of immersion, Figure 3a shows no observable mineral deposition on pristine PEEK, whereas p‐SPEEK, p/MBG‐SPEEK, and Mn@p/MBG‐SPEEK developed abundant globular Ca‐P aggregates, characteristic of amorphous calcium phosphate precursor phases that mediate hydroxyapatite formation [41, 42, 43]. This result indicated that sulfonation and subsequent modification substantially improved the in vitro bioactivity of PEEK. The chemical composition of these deposits was verified by FTIR (Figure 3b), where P‐O vibrational bands of PO_4_^3−^ near 500, 600, and 1030 cm^−1^ confirmed Ca‐P species deposition. Correspondingly, EDS results (Figure S3) revealed Ca and P exclusively on modified samples, and the Ca/P ratios (1.13–1.57) fell within the reported range of amorphous calcium phosphate (ACP), a metastable precursor phase that is recognized to transform into hydroxyapatite under physiological conditions [42, 43, 44, 45]. The XRD profiles (Figure 3c) further supported the amorphous nature of the deposited clusters. Compared with p‐SPEEK and p/MBG‐SPEEK, Mn@p/MBG‐SPEEK exhibited a moderately reduced amount of Ca‐P deposition. This effect may arise from Mn^2+^ competitively coordinating with catechol moieties, thereby partially reducing the number of Ca^2+^ nucleation sites [42]. The lower Ca/P ratio on Mn‐containing surfaces (1.13 ± 0.07) supported this interpretation. Importantly, ACP nucleation remained evident across all modified samples, indicating that Mn incorporation did not compromise the intrinsic mineralization capability of the modified surfaces. Taken together, these results confirm that the hybrid surface modifications successfully endowed PEEK with in vitro Ca‐P ‐forming capability, establishing a biomimetic Ca‐P interface for the subsequent biological evaluations.

### 2.4 Cytocompatibility and Osteogenic Regulation of Modified PEEK Surfaces

The biological compatibility and osteogenic modulation of the engineered PEEK surfaces were comprehensively evaluated in vitro to verify their biosafety and bone‐regenerative potential. MC3T3‐E1 pre‐osteoblasts were cultured on PEEK, p‐SPEEK, p/MBG‐SPEEK, and Mn@p/MBG‐SPEEK for 1 and 3 days, followed by CCK‐8 and live/dead fluorescence assays. As shown in Figure S4, all modified samples exhibited comparable cell viability to pristine PEEK at day 1, confirming their cytocompatibility and absence of acute toxicity. After 3 days, cell viability markedly increased in all groups, reflecting favorable proliferation support. Although a slightly lower proliferation was observed on p‐SPEEK, this mild reduction was likely associated with transient oxidative stimulation by the redox‐active catechol groups of PDA, which can elevate ROS levels within a non‐cytotoxic range [46]. In contrast, the incorporation of MBGNs significantly enhanced cell viability in p/MBG‐SPEEK and Mn@p/MBG‐SPEEK compared to p‐SPEEK, indicating that soluble Si‐containing species and Ca^2+^ release from MBGNs facilitated metabolic activity and adhesion of cells. The addition of Mn^2+^ at the designed concentration did not compromise cell viability, confirming its biosafety and potential therapeutic relevance. ICP‐OES results are presented in Figure S5a. Si species and Ca ions were gradually released from the MBGNs‐containing samples, while Mn was detected only from Mn@p/MBG‐SPEEK and showed a sustained release profile rather than rapid depletion, indicating a controlled release manner. In addition, the post‐immersion XPS and SEM‐EDS analyses (Figure S5b–f) further confirmed the stability of the Mn/PDA/MBGNs‐modified surfaces, together with ICP‐OES results suggesting a sustained biofunctional interface of Mn@p/MBG‐SPEEK. The live/dead assay (Figure 3d) further validated these findings. Predominant green fluorescence (Calcein‐AM) and minimal red fluorescence (PI) indicated that nearly all MC3T3‐E1 cells remained viable after 3 days of culture on the modified surfaces. All these results demonstrate that the modified PEEK samples maintain cytocompatibility and provide a supportive microenvironment for osteoblast proliferation, indicating their potential for orthopedic applications.

To further elucidate their osteogenic regulatory capacity, the expression of osteogenesis‐related genes, including alkaline phosphatase (ALP), runt‐related transcription factor 2 (Runx2), osteocalcin (OCN), and osteopontin (OPN), was quantified by real‐time PCR after 3 days of osteogenic induction (Figure 3e). The surface‐functionalized PEEK samples upregulated the expression of these genes to varying degrees. Compared to p‐SPEEK, subsequent incorporation of MBGNs and Mn ions progressively elevated the mRNA levels of ALP, OCN, OPN, and Runx2, with Mn@p/MBG‐SPEEK showing the most pronounced enhancement. Specifically, relative to PEEK, OCN expression increased 1.8 and 2.3 times in p/MBG‐SPEEK and Mn@p/MBG‐SPEEK, respectively, while OPN expression rose by 1.5‐ and 1.9‐fold. ALP expression in Mn@p/MBG‐SPEEK was approximately 1.2‐fold higher than that of PEEK. Since ALP activity represents early osteogenic differentiation, whereas OPN and OCN are late‐stage markers, these results suggest that MBGNs primarily promoted late‐stage osteogenic gene expression, while Mn ions contributed to both early and late differentiation stages [47, 48, 49]. These findings are consistent with previous reports demonstrating Mn‐mediated stimulation of osteogenic pathways [47, 50]. Together, the synergistic effects of therapeutic Mn^2+^ ions, bioactive soluble Si‐containing species and Ca^2+^ ions from MBGNs effectively enhanced osteogenic differentiation, highlighting the potential of Mn@p/MBG‐SPEEK as a multifunctional orthopedic implant material.

Since vascularization is essential for bone regeneration, the response of human umbilical vein endothelial cells (HUVECs) was evaluated [51, 52]. All samples showed acceptable endothelial cytocompatibility after 1 and 3 days of culture (Figure S6a). Mn@p/MBG‐SPEEK significantly upregulated VEGF and CD31 expression in HUVECs to approximately 1.30‐fold and 1.27‐fold of the PEEK group, respectively (Figure 3f), and promoted denser capillary‐like network formation in the tube formation assay, with mesh formation increasing to approximately 2.14‐fold of that of PEEK (Figure 3g; Figure S6c). The junction number and relative tube length exhibited similar increasing trends (Figure S6b,d). These results support the pro‐angiogenic potential of Mn@p/MBG‐SPEEK, which may be associated with soluble Si‐containing species and Ca^2+^ ions released from MBGNs and the presence of Mn^2+^ ions [53, 54, 55]. Together with the osteogenic results, the enhanced angiogenic response highlights the potential of Mn@p/MBG‐SPEEK for vascularized bone regeneration.

### 2.5 Mechanism Underlying Mn@p/MBG‐SPEEK‐Mediated Transcriptional Reprogramming and Osteogenic Commitment

To elucidate the molecular mechanism underlying the enhanced osteogenesis in MC3T3‐E1 cells cultured on Mn@p/MBG‐SPEEK, RNA sequencing was conducted to compare transcriptomic profiles across PEEK, p‐SPEEK, p/MBG‐SPEEK, and Mn@p/MBG‐SPEEK groups. As shown in the volcano plots (Figure 3h), p/MBG‐SPEEK induced the most extensive transcriptional alterations, with 78 differentially expressed genes (DEGs) relative to pristine PEEK, whereas p‐SPEEK and Mn@p/MBG‐SPEEK caused more moderate alterations. The hierarchical clustering heatmap (Figure S7a) revealed distinct gene‐expression signatures among the groups, indicating substrate‐specific transcriptional reprogramming. Gene Ontology (GO) categorization of DEGs (Figure S7b) demonstrated that Mn@p/MBG‐SPEEK primarily enriched pathways related to metabolic processes, biological regulation, and cellular anatomical entity, suggesting a transcriptional bias toward biosynthetic activation and metabolic remodeling [56, 57]. In contrast, p‐SPEEK and p/MBG‐SPEEK exhibited more substantial enrichment in immune response, stimulus perception, and cell locomotion.

Further GO enrichment analysis of upregulated DEGs (Figure 3i–k) revealed that Mn@p/MBG‐SPEEK promoted biological programs associated with vasculature development, response to growth factor, osteoblast proliferation, and extracellular matrix (ECM)‐related processes (collagen‐containing ECM, basement membrane, tenascin complex). Additionally, enrichment of AP‐1 transcription factor complex and RISC‐loading complex indicated activation of early osteogenic transcriptional regulators and miRNA‐mediated post‐transcriptional control. Molecular function enrichment for heparin, glycosaminoglycan, and syndecan binding pointed to enhanced ECM‐cell crosstalk and activation of growth factor‐responsive, mechanosensitive signaling cascades, in which syndecans potentiate integrin‐mediated adhesion and receptor signaling to drive matrix biosynthesis and osteogenic differentiation [58, 59]. The presence of the AP‐1 complex reflects early transcriptional priming for osteogenesis, whereas RISC‐associated regulation implies fine‐tuned modulation of metabolic and differentiation transcripts [60, 61, 62]. Consistent with these predictions, the Mn@p/MBG‐SPEEK group displayed upregulated ALP, Runx2, OCN, and OPN expression (Figure 3e), confirming the functional linkage between ECM sensing, growth factor response, and osteogenic gene activation. Integrative analysis of the GO and KEGG results (Figure S8) further indicated that Mn@p/MBG‐SPEEK activated ECM‐receptor interactions, PI3K/Akt signaling, focal adhesion, and hormone‐regulated pathways (e.g., growth hormone and GnRH signaling), all closely associated with osteogenic differentiation. Mechanistically, these cascades converge on integrin engagement and focal adhesion‐mediated activation of PI3K/Akt/mTOR and MAPK‐AP‐1 pathways, resulting in GSK3β phosphorylation, β‐catenin stabilization, and transcriptional upregulation of osteogenic regulators such as Runx2, ultimately sustaining high ALP and OCN expression [63, 64].

Overall, these findings delineate an integrated molecular network through which Mn@p/MBG‐SPEEK orchestrates osteogenic commitment. The system promotes ECM‐growth factor‐responsive signaling, coordinates transcriptional and post‐transcriptional regulation, and establishes a bioenergetic state suited for sustained osteoblast lineage differentiation. Compared to unmodified PEEK, the combined modification strategy, comprising PDA functionalization, MBGNs loading, and Mn chelation, reshapes the biochemical and mechanical cues at the cell‐material interface, thereby coupling angiogenic support, matrix signaling, and metabolic activation.

### 2.6 Photothermal and NIR‐Activated Antibacterial Performance

**FIGURE 4:**
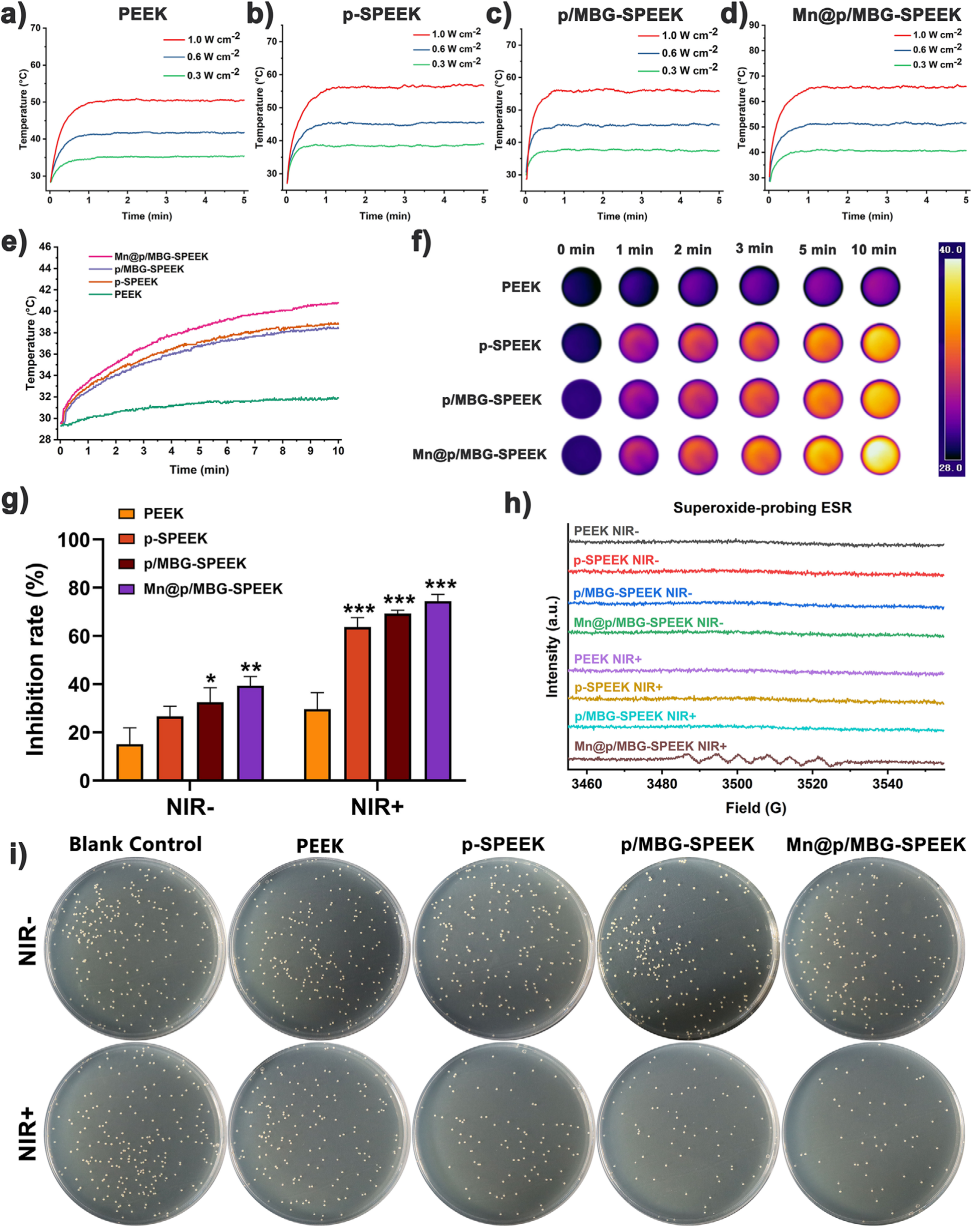
Photothermal properties and NIR‐mediated antibacterial activities of PEEK samples. (a–d) Photothermal curves of PEEK samples with different power densities of NIR laser at 0.3, 0.6, and 1.0 W cm^−2^ under dry conditions; (e) Real‐time infrared thermal images over time and (f) photothermal curves of PEEK samples in cell culture medium with NIR irradiation at 1.0 W cm^−2^. (g) Antibacterial effectiveness of various PEEK materials against *S. aureus* without and with NIR irradiation at 1.0 W cm^−2^ for 10 min (*n* = 3); (h) ESR spectra recorded under superoxide‐probing conditions without and with 808 nm NIR irradiation for 5 min in the presence of DMPO; (i) Corresponding images of *S. aureus* colonies growing on agar plates. Data are presented as mean ± SD. Statistical significance was analyzed by one‐way ANOVA followed by Tukey's HSD post hoc test. ^*^ *p* < 0.05, ^**^ *p* < 0.01, and ^***^ *p* < 0.001.

To further explore the therapeutic potential of the engineered PEEK surfaces, their photothermal behavior and NIR‐mediated antibacterial activity were systematically investigated. Upon 808 nm laser irradiation (0.3–1.0 W cm^−2^) under dry (air) conditions, all samples exhibited a rapid temperature increase within 1 min, followed by thermal stabilization (Figure 4a–d). The temperature rise was positively correlated with laser intensity, confirming controllable light‐to‐heat conversion. At a power density of 0.3 W cm^−2^, Mn@p/MBG‐SPEEK reached 40.7°C, exceeding PEEK (35.4°C), p‐SPEEK (38.2°C), and p/MBG‐SPEEK (37.8°C). A corresponding trend was also observed at higher laser power densities of 0.6 and 1.0 W cm^−2^. At 1.0 W cm^−2^, Mn@p/MBG‐SPEEK achieved 65.6°C within 1 min, markedly higher than PEEK (50.5°C) and other modified groups (∼56.0°C), revealing superior photothermal conversion capability. Infrared thermal imaging (Figure S9) further confirmed these results, showing a brighter thermal profile for Mn@p/MBG‐SPEEK. Under wet (cell‐culture medium) conditions, Mn@p/MBG‐SPEEK also exhibited efficient and stable photothermal performance (Figure 4e,f). After 10 min of irradiation at 1.0 W cm^−2^, its temperature increased by 11.3°C to 40.8°C, while PEEK displayed only a minor increase of ∼3°C. Given its short‐term and localized application, the NIR irradiation condition used here is expected to activate antibacterial efficacy while reducing the risk of severe thermal injury to surrounding normal cells, especially considering the moderate temperature reached under wet culture conditions, which is an essential prerequisite for potential clinical application. The improved light‐to‐heat conversion is attributed to Mn^2+^ coordination with PDA, which enhances π‐d charge transfer and increases PDA adsorption, leading to a thicker Mn@PDA layer and higher photothermal response [65, 66, 67, 68].

Based on these findings, the antibacterial capability of the PEEK samples was assessed against *Staphylococcus aureus* (*S. aureus*) under both dark and NIR‐irradiated conditions (Figure 4g,i). In the absence of irradiation, pristine PEEK showed a negligible antibacterial effect, whereas p‐SPEEK, p/MBG‐SPEEK, and Mn@p/MBG‐SPEEK exhibited modest antibacterial ratios of 26% ± 4%, 33% ± 6%, and 39% ± 4%, respectively. This inherent antibacterial activity originated mainly from the amphoteric and dynamic redox‐active features of PDA: protonated amine groups can interact electrostatically with negatively charged bacterial membranes, while PDA‐derived catechol/quinone‐related motifs may mediate electron transfer with O_2_ and induce ROS‐related oxidative stress, thereby disrupting bacterial integrity [46, 69, 70]. In addition, the incorporation of MBGNs further improved antibacterial capacity likely through localized pH elevation caused by Ca^2+^ release during degradation, creating an alkaline microenvironment hostile to bacteria [71]. Upon NIR irradiation (1.0 W cm^−2^ for 10 min), antibacterial efficacy was greatly enhanced, reaching 64% ± 4% for p‐SPEEK, 69% ± 1% for p/MBG‐SPEEK, and 74% ± 3% for Mn@p/MBG‐SPEEK. This pronounced improvement is attributed to the combined effects of localized hyperthermia and ROS‐involved oxidative stress. To experimentally support this ROS‐related pathway, electron spin resonance (ESR) measurements were performed under superoxide‐probing conditions. After 808 nm NIR irradiation for 5 min, Mn@p/MBG‐SPEEK exhibited a discernible multi‐peak ESR signal, whereas PEEK, p‐SPEEK, and p/MBG‐SPEEK showed negligible responses (Figure 4h). This result supports the NIR‐induced generation of superoxide‐related reactive species on Mn@p/MBG‐SPEEK. Additional ESR measurements under hydroxyl radical‐ and singlet oxygen‐probing conditions were also performed and results are provided in Figure S10. No obvious characteristic ESR signals were observed under these conditions, suggesting that the NIR‐induced ROS activity of Mn@p/MBG‐SPEEK was mainly associated with superoxide‐related reactive species. The heat produced by PDA can accelerate bacterial membrane disruption, while the temperature increase may facilitate redox reaction kinetics and amplify oxidative stress damage [46, 72]. Meanwhile, Mn^2+^ ions may reinforce bactericidal action through ion‐mediated mechanisms, including electrostatic disruption of bacterial membranes and interactions with thiol‐containing proteins, thereby suppressing bacterial replication and metabolic activity [20, 21, 73, 74]. Altogether, Mn@p/MBG‐SPEEK exhibited the most potent antibacterial effect under NIR irradiation, driven by the synergistic interplay amongIIT, photothermal therapy (PTT), and superoxide‐related ROS‐involved oxidative mechanisms. Thus this light‐responsive and multifunctional surface ensures both antibacterial protection and osteogenic compatibility, providing an effective platform for infection‐resistant bone regeneration. Importantly, when combined with the Mn‐induced transcriptional and metabolic reprogramming discussed in the preceding section, the proposed approach establishes a dual‐regulatory paradigm that integrates anti‐infective functionality with osteogenic activation, representing an attractive strategy for next‐generation orthopedic implants.

### 2.7 Evaluation of Mn‐Induced Optical and Electronic Structure Modulation

**FIGURE 5:**
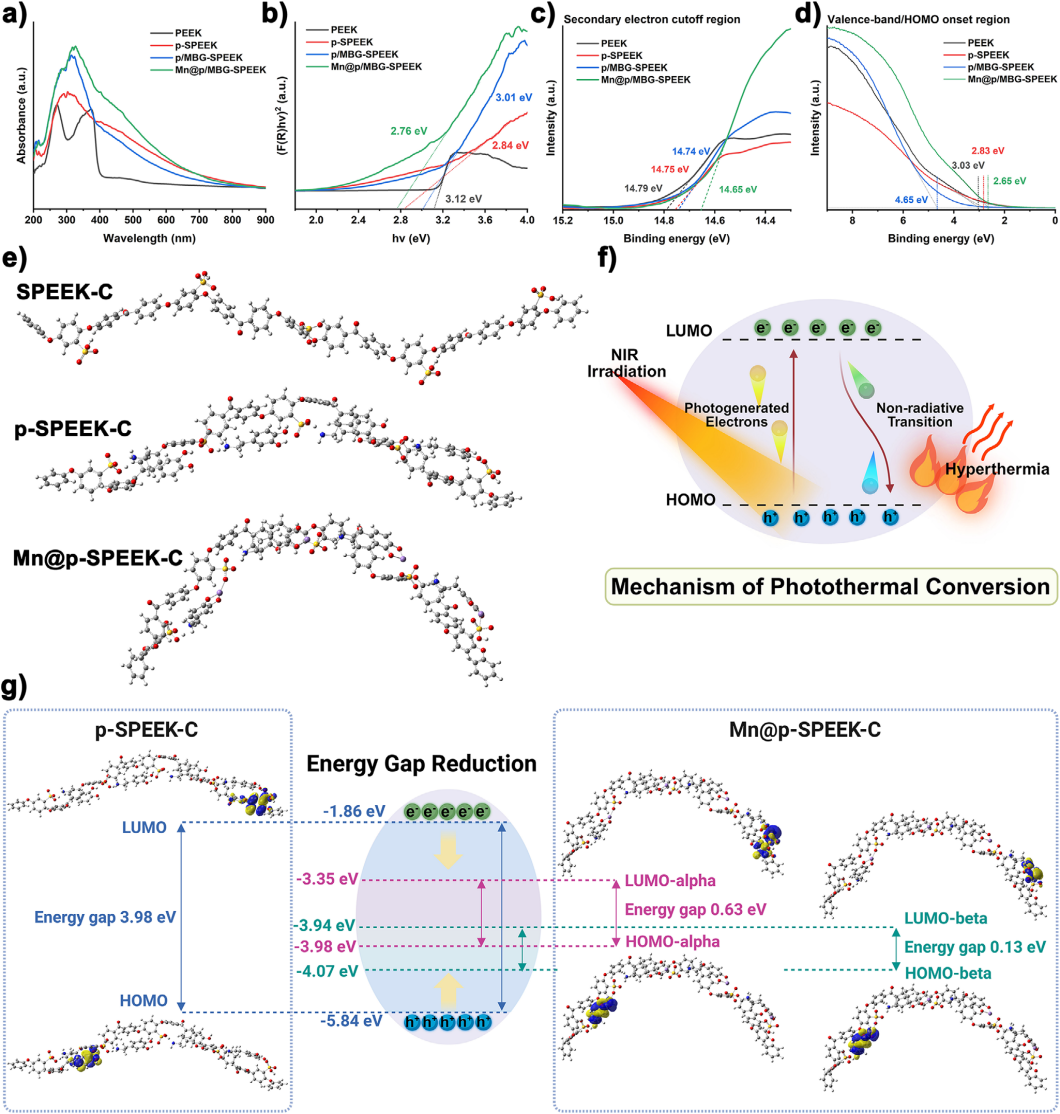
Optical and electronic structure characterization of PEEK‐based samples and DFT simulation of PEEK‐based systems. (a) UV–vis diffuse‐reflectance‐derived absorbance spectra of PEEK, p‐SPEEK, p/MBG‐SPEEK, and Mn@p/MBG‐SPEEK. (b) The corresponding Tauc plots and estimated optical bandgaps. (c,d) UPS spectra in the (c) secondary electron cutoff region and (d) valence‐band/HOMO onset region. (e) Optimized ground‐state geometries of SPEEK‐C, p‐SPEEK‐C, and Mn@p‐SPEEK‐C model clusters; (f) Schematic diagram of the photothermal conversion mechanism. (g) Theoretically calculated frontier molecular orbitals and HOMO‐LUMO energy gaps of p‐SPEEK‐C and Mn@p‐SPEEK‐C structures, showing Mn‐induced energy gap reduction.

To study the electronic structure modulation involved in the NIR‐triggered antibacterial mechanism, UV–vis diffuse‐reflectance analysis and ultraviolet photoelectron spectroscopy (UPS) measurements were performed. The UV–vis diffuse‐reflectance spectra (Figure 5a) showed that PDA deposition broadened the absorption of PEEK into the visible region, while MBGN incorporation decreased the absorption intensity compared with p‐SPEEK, likely due to the introduction of the weakly absorbing inorganic MBGNs and light‐scattering effects from the nanoparticles [75, 76]. Importantly, Mn@p/MBG‐SPEEK exhibited enhanced visible‐to‐NIR absorption compared with p/MBG‐SPEEK, indicating improved light‐harvesting capability after Mn coordination. The optical bandgap estimated from the Tauc plots decreased from 3.12 eV for PEEK to 2.84 eV for p‐SPEEK and further to 2.76 eV for Mn@p/MBG‐SPEEK, suggesting Mn‐induced narrowing of the optical gap (Figure 5b).

UPS measurements further revealed changes in the secondary electron cutoff and valence‐band/HOMO onset regions after surface modification of PEEK (Figure 5c,d). Mn@p/MBG‐SPEEK showed a distinct cutoff shift and altered valence‐band/HOMO onset compared with other samples, indicating that Mn coordination modulated the surface electronic states. Considering the electrically insulating nature of the PEEK substrate and the surface‐confined composite coating, the UPS‐derived values were used mainly for comparative assessment rather than absolute energy‐level determination.

Overall, the UV–vis spectra and UPS results provide experimental evidence that Mn coordination enhances visible‐to‐NIR absorption, narrows the optical gap, and alters the surface electronic structure of Mn@p/MBG‐SPEEK. These experimental findings are consistent with the DFT/TD‐DFT calculations discussed below, collectively supporting Mn‐induced energy‐gap narrowing and improving photoinduced charge‐transfer capability.

### 2.8 Theoretical Calculations Revealing the Mechanism of NIR‐Induced Antibacterial Efficacy

To further rationalize the experimentally observed optical/electronic structure modulation and the NIR‐triggered antibacterial performance of Mn@p/MBG‐SPEEK, DFT and TD‐DFT simulations were carried out. These calculations were designed to elucidate the molecular origin of the experimentally observed synergy between photothermal conversion and ROS‐involved oxidative antibacterial pathways. To the best of our knowledge, this is the first theoretical construction of PEEK‐based organic‐metallic hybrid complexes explicitly aimed at clarifying phototherapeutic mechanisms from fundamental principles. Because MBGNs did not markedly alter light‐to‐heat conversion in the above experiments, this inorganic phase was omitted from the models. Given the complex and debated polymerization of PDA, the dopamine monomer was adopted as a representative structural model, consistent with prior reports confirming its presence within PDA molecules [77, 78, 79, 80]. Model clusters were therefore built with at least four sulfonated PEEK (SPEEK) repeat units and either four dopamine (p) or four Mn‐chelated dopamine (Mn@p) molecules, yielding SPEEK‐C, p‐SPEEK‐C, and Mn@p‐SPEEK‐C (Figure S11). The stepwise transformations from SPEEK‐C to p‐SPEEK‐C and then to Mn@p‐SPEEK‐C exhibited increasingly negative Gibbs free energies, indicating spontaneous progression and enhanced thermodynamic stability. Mn@p‐SPEEK‐C was the most stable configuration, consistent with the higher density of Mn@PDA nanoparticles observed on Mn@p/MBG‐SPEEK (Figure 2b). Optimized ground‐state geometries are shown in Figure 5e. Consistent with the experimentally observed optical‐gap narrowing and UPS‐detected surface electronic modulation, frontier molecular orbital (FMO) analysis (Figure 5g) revealed a pronounced narrowing of the calculated HOMO‐LUMO energy gap upon Mn coordination, from ∼3.98 eV in p‐SPEEK‐C to 0.13 eV for the α‐orbitals and 0.63 eV for the β‐orbitals in Mn@p‐SPEEK‐C. Such energy gap reduction is consistent with the enhanced visible‐to‐NIR absorption observation and may facilitate photoactivation under NIR irradiation, thereby favoring non‐radiative relaxation conducive to heat generation [81, 82]. The conceptual light‐to‐heat pathway is sketched in Figure 5f. Mulliken spin population analysis (Figure S12) further showed that each Mn center bears a localized unpaired electron, with antiferromagnetic coupling across the four Mn sites. This spin arrangement stabilizes a symmetry‐broken singlet ground state while preserving local spin density that favors energy dissipation and electron transfer features beneficial for both photothermal and redox reactivity.

**FIGURE 6:**
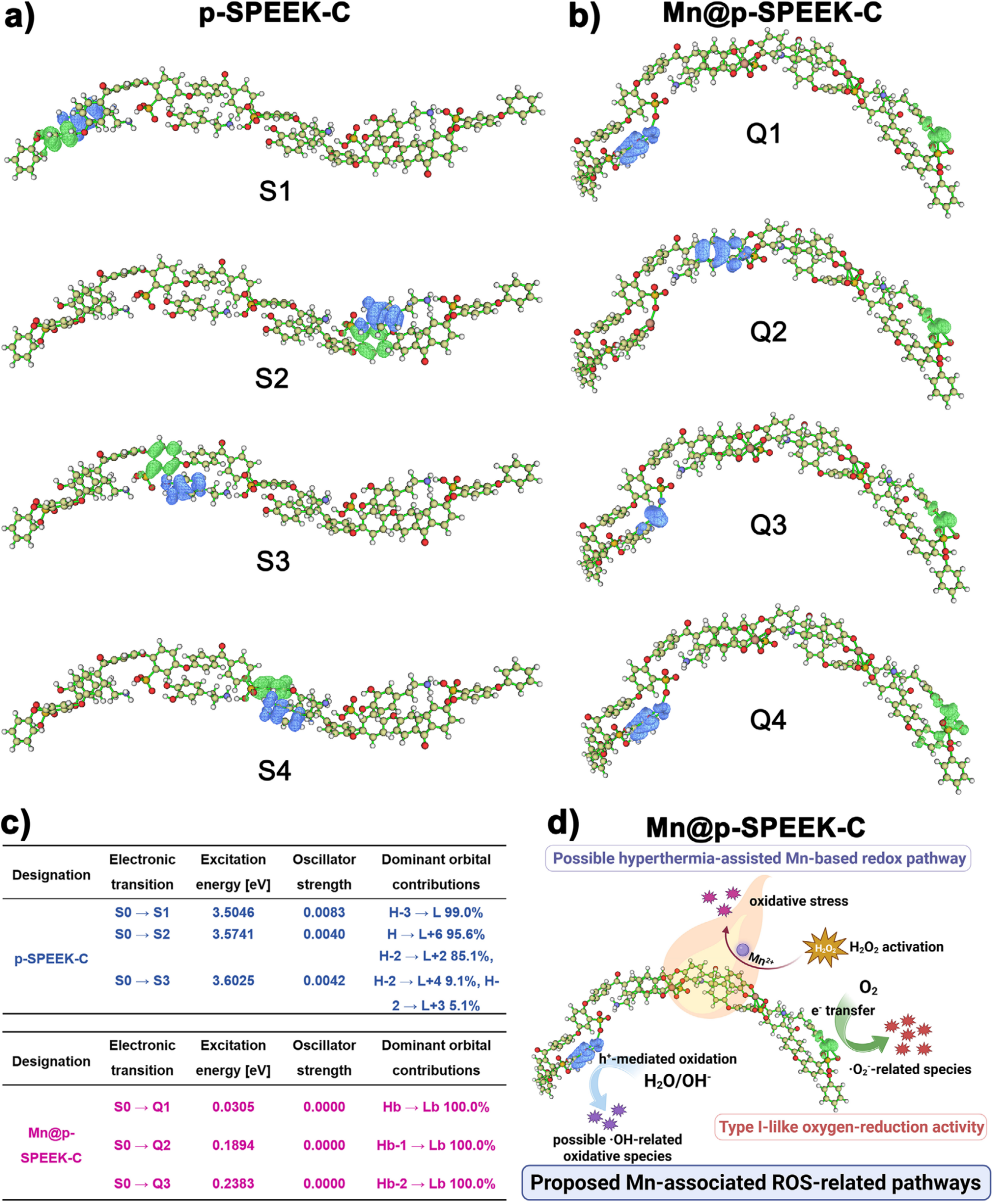
TD‐DFT simulations of excited‐state features in the PEEK‐based model systems. (a,b) The electron (green isosurface)‐hole (blue isosurface) density difference plots of (a) the first four singlet excited states (S1‐S4) in p‐SPEEK‐C and (b) the first four quintet excited states (Q1‐Q4) in Mn@p‐SPEEK‐C. (c) Representative TD‐DFT excited‐state results for the first three electronic transitions, including excitation energies, oscillator strengths, and dominant orbital contributions, of p‐SPEEK‐C and Mn@p‐SPEEK‐C. (d) Schematic illustration of the proposed Mn‐associated ROS‐related pathways in Mn@p‐SPEEK‐C under photoactivation.

TD‐DFT simulations were further used to examine the excited‐state features that may favor ROS‐related redox processes, as partially shown in Figure 6c, with full summaries listed in Tables S2 and S3. Mn@p‐SPEEK‐C showed multiple quintet excitations (Q1‐Q10) with negligible oscillator strengths, consistent with strong Mn‐mediated spin‐orbit coupling that facilitates intersystem crossing (ISC) [83]. By comparison, p‐SPEEK‐C underwent singlet ground state (S0) to singlet excited states (from S1–S12) and also to different triplet excited states (from T1 to T5) [83]. Triplet excited states are generally regarded as important intermediates in photosensitized ROS‐related processes [84]. In addition, Mn chelation lowered excitation energies and red‐shifted the absorption relative to p‐SPEEK‐C, indicating that lower‐energy photons under biologically relevant NIR irradiation suffice to drive electronic excitation. Enhanced orbital interactions together with a narrowed energy gap may prolong the lifetimes of charge carriers (electrons and holes), favoring both non‐radiative relaxation (heat production) and ROS‐involved redox activity [85, 86, 87]. Electron–hole density‐difference mappings provided direct evidence for charge‐separation behavior, with the first four excited states shown in Figure 6a,b and the remaining excited states summarized in Figures S13 and S14. In p‐SPEEK‐C, electron and hole densities substantially overlapped, which could promote recombination. In contrast, in Mn@p‐SPEEK‐C, electrons were predominantly localized near Mn centers, whereas holes resided on dopamine units. This spatial separation suppresses recombination losses and prolongs carrier lifetimes. Mechanistically, Mn probably acts as an electron reservoir that facilitates photoexcited electron transfer to molecular oxygen, thereby promoting Type I‐like photodynamic (oxygen‐reduction) pathways and generation of superoxide‐related reactive species (Figure 6d) [88, 89, 90, 91, 92]. Meanwhile, photogenerated holes may participate in oxidative reactions involving water or hydroxide species, potentially contributing to broader oxidative processes (Figure 6d). Local hyperthermia generated by photothermal conversion may also assist Mn‐associated redox reactions involving H_2_O_2_ activation, which could contribute to the overall oxidative stress microenvironment (Figure 6d). PDA catechol groups may act as endogenous reductants to support Mn^3+^/Mn^2+^ redox cycling, thereby sustaining Mn‐associated redox activity and amplifying antibacterial oxidative damage [22, 93]. In sum, Mn chelation lowers excitation thresholds and narrows the electronic gap of Mn@p‐SPEEK‐C, enabling dual activation: efficient heat release through non‐radiative relaxation and ROS‐involved redox activity via spin‐enabled excited‐state pathways. The result is a synergistic antibacterial effect in which hyperthermia and oxidative stress jointly compromise bacterial membranes and metabolic function.

Collectively, the computational evidence outlines a coherent molecular mechanism that accounts for the superior antibacterial performance of Mn@p‐SPEEK‐C. The convergence of photothermal effects with Type I‐like ROS‐related redox pathways and possible hyperthermia‐assisted chemodynamic processes offers a unified mechanistic framework for engineering light‐responsive biomaterials capable of clinically relevant disinfection while maintaining biocompatibility and osteogenic potential.

## 3 Conclusions

A multifunctional Mn@p/MBG‐SPEEK biomaterial was developed to address implant‐related infections by integrating osteogenic and angiogenic stimulation with light‐triggered antibacterial capability. The synergistic combination of PDA nanolayering, mesoporous bioactive glass nanoparticles, and Mn‐ion chelation endowed PEEK with enhanced hydrophilicity, hierarchical porosity, roughness, and stable bioactive surfaces without compromising its physicochemical integrity. The modified substrates promoted amorphous calcium phosphate deposition and upregulated osteogenic markers (ALP, Runx2, OCN, OPN) through activation of PI3K/Akt/mTOR and AP‐1 pathways, confirming osteoinductive activity. The angiogenic assessment further demonstrated the pro‐angiogenic potential of Mn@p/MBG‐SPEEK, suggesting its ability to promote vascularized bone regeneration. Under NIR irradiation, Mn@p/MBG‐SPEEK exhibited efficient photo‐to‐thermal conversion and strong antibacterial efficacy against *S. aureus* via synergistic photothermal effects, superoxide‐related ROS‐involved oxidative stress, Mn‐associated redox and ion‐mediated mechanisms. UV–vis, UPS, ESR, and DFT/TD‐DFT analyses collectively revealed that Mn coordination modulated the optical/electronic structure, promoted electron–hole separation, and favored ROS‐related redox pathways. Overall, this work establishes a multifunctional and mechanistically guided ion‐polymer surface modification strategy for designing next‐generation PEEK implants that combine durable antibacterial defense with osteogenic and angiogenic regeneration.

## 4 Experimental Section

### 4.1 Preparation of MBGNs

MBGNs with a nominal composition of 70SiO_2_‐30CaO (in mol%) were synthesized by using a microemulsion‐based sol–gel method. Briefly, 2.8 g of cetyltrimethylammonium bromide (CTAB) was dissolved in 132 mL of deionized water (DI water) under continuous stirring. Then 40 mL of ethyl acetate (EA) was added and stirred for 30 min followed by the addition of 28 mL of aqueous ammonia (1m, diluted from 28 wt.% ammonium hydroxide). After 15 min, 14.4 mL of tetraethyl orthosilicate (TEOS) and 6.5 g of calcium nitrate tetrahydrate (CN) were sequentially added to the above mixture in an interval of 30 min. After further reaction for 4 h, the resulted nanoparticles were collected and washed twice with DI water and once with ethanol by centrifugation. The collected deposits were eventually dried at 60°C overnight prior to calcination at 700°C for 3 h with a heating rate of 2°C min^−1^. All used chemicals were purchased from Sigma–Aldrich (Darmstadt, Germany) and used without further purification.

### 4.2 Fabrication of Surface Functionalized PEEK Samples

In this research, commercially available medical PEEK rods (TECAPEEK MT natural, Ensinger, Germany) were machined into discs with dimensions of Φ 12 × 2 mm for all studies. After being carefully polished using SiC sandpapers with progressively finer grits, these PEEK samples were washed ultrasonically in ethanol, acetone, and DI water successively, then dried in an oven for further experiments. The sulfonated PEEK samples (SPEEK) were produced by immersing clean PEEK discs into concentrated sulfuric acid (95‐97 wt.%) under magnetic stirring for 5 min. After that, the samples were cleaned with DI water and then hydrothermally treated at 100°C for 4 h to thoroughly remove any sulfur residues [34]. Afterward, the SPEEK samples were incubated in dopamine hydrochloride (2 mg mL^−1^) dissolved in Tris‐HCl buffer solution (10 mm, pH = 8.5) with or without the addition of MBGNs (1 mg mL^−1^) for 16 h in an orbital shaker at 200 rpm in dark to obtain PDA surface‐modified SPEEK samples (p‐SPEEK) and PDA/MGBNs surface‐modified SPEEK (p/MBG‐SPEEK). Thereafter, manganese (II) chloride tetrahydrate with a concentration of 0.625 mg mL^−1^ was added into the PDA/MBGNs Tris‐HCl buffer suspension for another 2 h to obtain Mn^2+^ chelated p/MBG‐SPEEK samples (Mn@p/MBG‐SPEEK). The MnCl_2_·4H_2_O concentration was selected based on previous studies with appropriate modification and further optimized by preliminary cytotoxicity tests using Mn@p/MBG‐SPEEK samples prepared with different MnCl_2_·4H_2_O concentrations [94, 95]. Finally, all these surface‐functionalized PEEK samples were washed twice with DI water and once with ethanol under ultrasonication, and then they were dried in the oven at 60°C overnight. All chemicals were purchased from Sigma–Aldrich (Darmstadt, Germany) and were used without further purification.

### 4.3 Physicochemical Characterization of MBGNs and PEEK‐Based Samples

Scanning‐transmission electron microscopy ((S)TEM, FEI Talos F200S, Thermo Fisher Scientific, US), including high‐angle annular dark field (HAADF) imaging and super‐X energy dispersive X‐ray spectrometry (EDS), was employed to characterize the microstructure and elemental distribution of MBGNs. In addition, field‐emission scanning electron microscopy (FE‐SEM, Auriga, Carl Zeiss, Germany) equipped with an energy dispersive X‐ray spectroscopy (EDS, X‐MaxN, Oxford Instruments, UK) system was performed to analyze their surface morphology and elemental composition. The average particle size and particle size distribution of MBGNs were determined by ImageJ software (National Institutes of Health, Bethesda, MD, USA), and more than 200 particles were measured. Nitrogen adsorption‐desorption analysis was conducted on a porosimeter (ASAP2460, Micrometrics Instrument) at 77.35 K to study the textural properties of MBGNs. The specific surface area (SSA) of MBGNs was obtained by the Brunauer‐Emmett‐Teller (BET) method. The Barrett‐Joyner‐Halenda (BJH) analysis method was used to estimate the cumulative pore volume and pore size distribution of MBGNs. The chemical composition of MBGNs was quantitatively determined by inductively coupled plasma optical emission spectroscopy (ICP‐OES, Agilent 5100, Agilent Technologies) after appropriate sample preparation. The measured Si and Ca concentrations were converted into the corresponding oxide contents, namely SiO_2_ and CaO, and expressed as molar percentages. The surface chemical composition of MBGNs and PEEK samples was further investigated by X‐ray photoelectron spectroscopy (XPS, Nexsa G2, Thermo Fisher Scientific Inc.). The surface morphology and chemical composition of PEEK samples, both before and after functionalization, were studied using FE‐SEM, EDS spectra, and mapping analysis. Fourier transform infrared spectroscopy (FTIR, IRAffinity‐1S, SHIMADZU, Japan) and X‐ray diffraction analysis (XRD, MiniFlex 600, Rigaku, Japan) were performed to investigate the composition and structure of samples. Simultaneous thermal analysis (STA, Setaram Setsys Evolution, France) was employed to analyze the thermal stability of samples from room temperature to 850°C. It provided information about endothermic and exothermic reactions as well as the weight change of PEEK‐based samples. All thermal experiments were conducted using approximately 11–16 mg of samples in an alumina crucible at a constant heating rate of 10 K min^−1^ in an argon atmosphere with a continuous flow rate. The wettability of PEEK samples was determined by using contact angle measurements (DSA30, Kruess GmbH, Germany). The surface roughness was evaluated using a 3D laser scanning confocal microscope (LEXT OLS5000, Olympus Co., Shinjuku, Japan) over an area of 25 µm^2^. Ultraviolet‐visible spectrometer (UV–vis, Shimadzu, UV‐3600, Kyoto, Japan) with a diffuse reflectance accessory and ultraviolet photoelectron spectroscopy (UPS, Nexsa G2, Thermo Fisher Scientific Inc.) were used to evaluate the optical absorption behavior and surface electronic structure of the PEEK‐based samples.

### 4.4 In Vitro Bioactivity

The acellular bioactivity of the specimens was investigated in vitro through the examination of mineral precipitation after immersion in simulated body fluid (SBF), following the procedure proposed by Kokubo [40]. In brief, four groups of PEEK samples were soaked in SBF in an orbital shaker at a speed of 90 rpm and a temperature of 37°C for up to one week. At the determined time point, the samples were retrieved and rinsed with DI water prior to being dried at 60°C in an electric oven overnight. The mineralization of the sample surfaces was subsequently assessed by FTIR, XRD, and SEM‐EDS analyses.

### 4.5 Cell Culture Medium Immersion Study

To evaluate biofunctional component release and surface stability, different PEEK samples were immersed in 2 mL of α‐MEM (HyClone, USA) at 37°C under orbital shaking for up to 7 days, with three replicates per group. At days 1, 3, and 7, the extracts were collected and replaced with 2 mL of fresh α‐MEM. Then, the extracts were analyzed by ICP‐OES to determine cumulative Si, Ca, and Mn release. After 7 days, the immersed samples were characterized by SEM‐EDS and XPS to assess surface morphology, elemental distribution, and chemical composition.

### 4.6 In Vitro Cytocompatibility Evaluation With MC3T3‐E1 Cells

Pre‐osteoblasts MC3T3‐E1 cells were obtained from the Chinese Academy of Sciences Cell Bank (Shanghai, China) and utilized in this study. The MC3T3‐E1 cells were cultured in α‐MEM (Hyclone, USA) supplemented with 10% fetal bovine serum (Gibco, USA), 100 U mL^−1^ penicillin, and 100 µg mL^−1^ streptomycin (Hyclone, USA). MC3T3‐E1 cells were seeded at a density of 3 × 10^4^ cells per sample on different PEEK samples in separate 24‐well culture plates. After 1 and 3 days, the viability of MC3T3‐E1 cells co‐cultured with a series of PEEK samples was determined using a Cell Counting Kit‐8 (CCK‐8) assay (Beyotime, Shanghai, China). After that, 3‐day co‐cultured cells of each group were stained with 4,6‐diamidino‐2‐phenylindole (DAPI) and calcein‐AM/propidium iodide (PI) cytotoxicity assay kit (Beyotime, China) according to the manufacturer's instructions. The cells were washed with PBS and observed using the Cytation 3 Cell Imaging Multi‐Mode Reader (Agilent Technologies, USA).

### 4.7 In Vitro Osteogenesis

The in vitro osteogenic differentiation potential of MC3T3‐E1 cells in response to a series of PEEK samples was determined using quantitative real‐time PCR (qRT‐PCR) analysis. Briefly, total RNA was isolated from MC3T3‐E1 cells after 3 days of co‐culture with the samples using the RNeasy Mini Kit (QiAGEN, Germany) in accordance with the manufacturer's instructions. Subsequently, complementary DNA (cDNA) was synthesized from the purified RNA using the PrimeScript RT reagent Kit (Takara, Japan). Quantitative real‐time PCR (qRT‐PCR) analysis was conducted with SYBR Premix EX Taq (Takara, Japan) on a LightCycler 96 system (Roche, Switzerland) to assess gene expression levels. The primer sequences used are listed in Table S4.

### 4.8 Transcriptome Sequencing Analysis

Total RNA was extracted from MC3T3‐E1 cells treated with the four groups of PEEK samples for 3 days using TRIzol Reagent (ThermoFisher, USA). The extracted RNA was used to synthesize complementary DNA (cDNA), and libraries were subsequently prepared following standard high‐throughput sequencing protocols. After end‐repair and PCR amplification steps, the size distribution and integrity of the cDNA libraries were assessed using the Agilent 2100 Bioanalyzer, while their concentrations were quantified via a real‐time PCR system (ABI StepOnePlus, USA). High‐quality reads were obtained by filtering out low‐quality sequences and adapter contaminants. Clean reads were then aligned to the reference genome using a splice‐aware alignment method. Additional alignment to the reference gene sequences was performed to enhance mapping accuracy. Gene expression levels were quantified using an established transcript‐level estimation algorithm designed for RNA‐Seq data. Differential gene expression was determined using statistical modeling in R, with significance thresholds set at an adjusted *p*‐value < 0.05 and a fold change greater than 2. Identified differentially expressed genes were subjected to Gene Ontology (GO) enrichment analysis to determine associated biological processes and molecular functions. Furthermore, pathway‐level insights were gained through KEGG‐based analysis to explore signaling pathways that the different PEEK samples may regulate.

### 4.9 In Vitro Endothelial Cell Cytocompatibility and Angiogenic Evaluation

Human umbilical vein endothelial cells (HUVECs) and green fluorescent protein‐labeled HUVECs (HUVECs‐GFP) were obtained from Cellverse Bioscience Technology Co., Ltd. (Shanghai, China). HUVECs were directly seeded onto different PEEK samples to evaluate endothelial cell cytocompatibility and angiogenesis‐related gene expression. Cell viability was assessed after 1 and 3 days using the CCK‐8 assay as described above. For qRT‐PCR analysis, total RNA was extracted from HUVECs after 3 days of direct culture on the samples, followed by cDNA synthesis and quantitative amplification using the same protocol described above. The expression levels of VEGF and CD31 were normalized to GAPDH, and the primer sequences are listed in Table S5. For the tube formation assay, extracts of different PEEK samples were prepared by immersing each sample in 2 mL of complete endothelial cell culture medium at 37°C under shaking at 60 rpm. HUVECs‐GFP were seeded onto Matrigel‐coated plates and cultured with the corresponding extracts. After 6 h of incubation, capillary‐like network formation was imaged under a fluorescence microscope. The number of junctions, number of meshes, and relative tube length were quantified using ImageJ software.

### 4.10 Photothermal Responsiveness

The photothermal performance of the PEEK samples was determined using an infrared thermal imaging system (FOTRIC 220s, China). In this experiment, diverse samples were placed into a 24‐well plate and irradiated with a near‐infrared (NIR) laser (Shanghai Connect Fiber Optics Company, Shanghai, China) with a wavelength of 808 nm and power densities of 0.3, 0.6, and 1.0 W cm^−2^ for 5 min. To study the thermal properties under wet conditions, samples were immersed in cell culture medium within a 24‐well plate and exposed to the NIR laser at 808 nm (1.0 W cm^−2^) for 10 min. The variations in temperature and IR images were recorded by an infrared camera (FOTRIC 220s, China).

### 4.11 ROS‐Related Response Evaluation

Electron spin resonance measurements (ESR, Bruker EMXplus X‐band EPR spectrometer, Karlsruhe, Germany) were performed to evaluate NIR‐induced ROS‐related signals from the PEEK‐based samples. Briefly, the samples were immersed in the corresponding probe solution and irradiated with and without an 808 nm NIR laser for 5 min. After irradiation, the solution was immediately collected and transferred into quartz capillary tubes for ESR analysis. Superoxide‐related reactive species were detected using 5,5‐dimethylpyrroline N‐oxide (DMPO, Sigma–Aldrich, USA), while hydroxyl radical‐ and singlet oxygen‐probing measurements were performed using DMPO and 2,2,6,6‐tetramethylpiperidine (TEMP, 95%, Sigma–Aldrich, USA), respectively.

### 4.12 In Vitro Antibacterial Assays

*Staphylococcus aureus* (ATCC 29213) was used as the bacterial model and cultured in Luria‐Bertani (LB) medium (Sango, China) under standard conditions. To evaluate the photothermal antibacterial performance of different types of PEEK samples, a bacterial suspension of *S. aureus* was prepared at a concentration of 1 × 10^6^ CFU mL^−1^ in the LB medium. Then, 200 µL of this suspension was carefully applied to the surface of each sample without or with NIR irradiation (1.0 W cm^−2^) for 10 min. After treatment, the bacterial suspensions were diluted 10‐fold to a series of concentrations and incubated on LB agar plates at 37°C for 18 h. After incubation, colony‐forming units (CFUs) were photographed and counted to assess antibacterial activity. The killing ratio was calculated using the following equation:

(1) $$Antibacterial\;rate\;\;\left(\%\right)=\frac{1-T}{C}\;\times 100\%$$

where T and C denote the bacterial colony counts (CFU per sample) for the test group (PEEK, p‐SPEEK, p/MBG‐SPEEK Mn@p/MBG‐SPEEK) and the blank control group, respectively.

### 4.13 DFT and TD‐DFT Theoretical Calculations

All DFT and TD‐DFT calculations were carried out with Gaussian 16. Ground‐state geometries were fully optimized in the gas phase with the PBE0‐D3BJ functional and the def2‐SVP basis set, and were confirmed as minima by the absence of imaginary frequencies. This level of theory has been shown to be robust for a variety of organometallic complexes [96]. Single‐point energies were subsequently refined at PBE0‐D3BJ/def2‐TZVP on the previously optimized geometries to obtain more accurate electronic energies. TD‐DFT calculations were computed at the TD‐PBE0/def2‐SVP level using the ground‐state structures. Spin‐density distributions, electron–hole maps, and natural transition orbitals were analyzed with Multiwfn 3.8 (dev).

### 4.14 Statistical Analysis

All experiments were conducted with at least three independent replicates unless otherwise stated. The results are presented as mean values ± standard deviation (SD). Statistical analyses were performed using one‐way or two‐way analysis of variance (ANOVA) followed by Tukey's Honestly Significant Difference (HSD) post hoc test, as appropriate for the experimental design. When the *p*‐value was less than 0.05, the differences were considered statistically significant.

## Author Contributions

**Zhiyan Xu**: conceptualization; formal analysis; investigation; validation; methodology; visualization; Writing – original draft preparation; Writing – review & editing. **Ondřej Adam**: formal analysis; investigation; writing – review & editing. **Marek Doubrava**: formal analysis; writing – review & editing. **Ana M. Beltrán**: formal analysis; writing – review & editing. **Ivo Dlouhý**: methodology; resources; funding acquisition; supervision; writing – review & editing. **Xin Liu**: investigation; methodology; formal analysis; supervision; resources; funding acquisition; writing – review & editing. **Baiyan Sui**: conceptualization; formal analysis; investigation; validation; methodology; resources; supervision; writing – original draft preparation; writing – review & editing. **Aldo R. Boccaccini**: conceptualization; project administration; supervision; resources; funding acquisition; writing – review & editing.

## Funding

The China Scholarship Council (CSC, No. 202006740028); Shanghai Pujiang Programme (23PJD050); National Natural Science Foundation of China (31900956 and 82271024); Science and Technology Commission of Shanghai Municipality (21DZ2291700); The European Virtual Institute on Knowledge‐Based Multifunctional Materials AISBL (KMM‐VIN) Research Fellowship (call 14, 2022).

## Conflicts of Interest

The authors declare no conflicts of interest.

## Supporting information




**Supporting File**: adma73945‐sup‐0001‐SuppMat.docx.

## Data Availability

The data that support the findings of this study are available from the corresponding author upon reasonable request.

## References

[1] H. Ma , A. Suonan , J. Zhou , et al., “PEEK (Polyether‐ether‐ketone) and Its Composite Materials in Orthopedic Implantation,” Arabian Journal of Chemistry 14 (2021): 102977, 10.1016/j.arabjc.2020.102977.

[2] J. R. Dondani , J. Iyer , and S. D. Tran , “Surface Treatments of PEEK for Osseointegration to Bone,” Biomolecules 13 (2023): 464, 10.3390/biom13030464. 36979399 PMC10046336

[3] S. Najeeb , Z. Khurshid , S. Zohaib , and M. S. Zafar , “Bioactivity and Osseointegration of PEEK Are Inferior to Those of Titanium: A Systematic Review,” Journal of Oral Implantology 42 (2016): 512–516, 10.1563/aaid-joi-D-16-00072. 27560166

[4] A. Schwitalla and W.‐D. Müller , “PEEK Dental Implants: A Review of the Literature,” Journal of Oral Implantology 39 (2013): 743–749, 10.1563/AAID-JOI-D-11-00002. 21905892

[5] N. Limaye , L. Veschini , and T. Coward , “Assessing Biocompatibility & Mechanical Testing of 3D‐printed PEEK versus Milled PEEK,” Heliyon 8 (2022): e12314, 10.1016/j.heliyon.2022.e12314. 36590483 PMC9800332

[6] Z. Zheng , P. Liu , X. Zhang , et al., “Strategies to Improve Bioactive and Antibacterial Properties of Polyetheretherketone (PEEK) for Use as Orthopedic Implants,” Materials Today Bio 16 (2022): 100402, 10.1016/j.mtbio.2022.100402. PMC9466655 36105676

[7] S. Akay and A. Yaghmur , “Recent Advances in Antibacterial Coatings to Combat Orthopedic Implant‐Associated Infections,” Molecules 29 (2024): 1172, 10.3390/molecules29051172. 38474684 PMC10935003

[8] P. Feng , R. He , Y. Gu , F. Yang , H. Pan , and C. Shuai , “Construction of Antibacterial Bone Implants and Their Application in Bone Regeneration,” Materials Horizons 11 (2024): 590–625, 10.1039/D3MH01298K. 38018410

[9] X. Kong , X. Zhang , Y. Wang , and B. Zhang , “Current Advances of Antibacterial Biomaterials for Bone Regeneration,” Advanced Materials Interfaces 12 (2025): 2400960, 10.1002/admi.202400960.

[10] D. Huo , F. Wang , F. Yang , et al., “Medical Titanium Surface‐modified Coatings With Antibacterial and Anti‐adhesive Properties for the Prevention of Implant‐associated Infections,” Journal of Materials Science & Technology 179 (2024): 208–223, 10.1016/j.jmst.2023.09.016.

[11] Y. Deng , J. Shi , Y. K. Chan , et al., “Heterostructured Metal–Organic Frameworks/Polydopamine Coating Endows Polyetheretherketone Implants With Multimodal Osteogenicity and Photoswitchable Disinfection,” Advanced Healthcare Materials 11 (2022): 2200641, 10.1002/adhm.202200641. 35521819

[12] Y. Chen , Y. Gao , Y. Chen , L. Liu , A. Mo , and Q. Peng , “Nanomaterials‐based Photothermal Therapy and Its Potentials in Antibacterial Treatment,” Journal of Controlled Release 328 (2020): 251–262, 10.1016/j.jconrel.2020.08.055. 32889053

[13] Y. Yang , L. Ma , C. Cheng , et al., “Nonchemotherapic and Robust Dual‐Responsive Nanoagents With On‐Demand Bacterial Trapping, Ablation, and Release for Efficient Wound Disinfection,” Advanced Functional Materials 28 (2018): 1705708, 10.1002/adfm.201705708.

[14] H. Xu , Y. Zhang , H. Zhang , et al., “Smart Polydopamine‐based Nanoplatforms for Biomedical Applications: State‐of‐art and Further Perspectives,” Coordination Chemistry Reviews 488 (2023): 215153, 10.1016/j.ccr.2023.215153.

[15] Y. Liu , K. Ai , J. Liu , M. Deng , Y. He , and L. Lu , “Dopamine‐Melanin Colloidal Nanospheres: An Efficient Near‐Infrared Photothermal Therapeutic Agent for In Vivo Cancer Therapy,” Advanced Materials 25 (2013): 1353–1359, 10.1002/adma.201204683. 23280690

[16] H. Lee , S. M. Dellatore , W. M. Miller , and P. B. Messersmith , “Mussel‐Inspired Surface Chemistry for Multifunctional Coatings,” Science 318 (2007): 426–430, 10.1126/science.1147241. 17947576 PMC2601629

[17] H.‐H. Lu , D. Ege , S. Salehi , and A. R. Boccaccini , “Ionic Medicine: Exploiting Metallic Ions to Stimulate Skeletal Muscle Tissue Regeneration,” Acta Biomaterialia 190 (2024): 1–23, 10.1016/j.actbio.2024.10.033. 39454933

[18] Z. Wang , Y. Zou , Y. Li , and Y. Cheng , “Metal‐Containing Polydopamine Nanomaterials: Catalysis, Energy, and Theranostics,” Small 16 (2020): 1907042, 10.1002/smll.201907042. 32220006

[19] G. Taskozhina , G. Batyrova , G. Umarova , Z. Issanguzhina , and N. Kereyeva , “The Manganese–Bone Connection: Investigating the Role of Manganese in Bone Health,” Journal of Clinical Medicine 13 (2024): 4679, 10.3390/jcm13164679. 39200820 PMC11355939

[20] Q. Nawaz , M. A. Ur Rehman , A. Burkovski , et al., “Synthesis and Characterization of Manganese Containing Mesoporous Bioactive Glass Nanoparticles for Biomedical Applications,” Journal of Materials Science: Materials in Medicine 29 (2018): 64, 10.1007/s10856-018-6070-4. 29737411

[21] S. Muthusamy , B. Mahendiran , S. Sampath , S. N. Jaisankar , S. K. Anandasadagopan , and G. S. Krishnakumar , “Hydroxyapatite Nanophases Augmented With Selenium and Manganese Ions for Bone Regeneration: Physiochemical, Microstructural and Biological Characterization,” Materials Science and Engineering: C 126 (2021): 112149, 10.1016/j.msec.2021.112149. 34082960

[22] W. Xu , B. Sun , F. Wu , et al., “Manganese Single‐atom Catalysts for Catalytic‐photothermal Synergistic Anti‐infected Therapy,” Chemical Engineering Journal 438 (2022): 135636, 10.1016/j.cej.2022.135636.

[23] C. Jia and F.‐G. Wu , “Antibacterial Chemodynamic Therapy: Materials and Strategies,” BME Frontiers 4 (2023): 0021, 10.34133/bmef.0021. 37849674 PMC10351393

[24] L. L. Hench , “The Story of Bioglass®,” Journal of Materials Science: Materials in Medicine 17 (2006): 967–978, 10.1007/s10856-006-0432-z. 17122907

[25] S. Pourshahrestani , E. Zeimaran , C. Janko , et al., “The Effect of Mesoporous Bioactive Glass Nanoparticles Incorporating Various Metallic Ions (Cu, Zn, Mn, Te) on wound healing,” Materials Advances 5 (2024): 6630–6647, 10.1039/D4MA00392F.

[26] C.‐M. Huo , L. Chen , H.‐Y. Wang , et al., “Density Functional Theory‐guided Drug Loading Strategy for Sensitized Tumor‐homing Thermotherapy,” Chemical Engineering Journal 423 (2021): 130146, 10.1016/j.cej.2021.130146.

[27] A. dos Santos da Silva and J. H. Z. dos Santos , “Stöber Method and Its Nuances Over the Years,” Advances in Colloid and Interface Science 314 (2023): 102888, 10.1016/j.cis.2023.102888. 37001206

[28] D. Altan , A. C. Özarslan , C. Özel , K. Tuzlakoğlu , Y. M. Sahin , and S. Yücel , “Fabrication of Electrospun Double Layered Biomimetic Collagen–Chitosan Polymeric Membranes With Zinc‐Doped Mesoporous Bioactive Glass Additives,” Polymers (Basel) 16 (2024): 2066, 10.3390/polym16142066. 39065383 PMC11281005

[29] K. Zheng , E. Torre , A. Bari , et al., “Antioxidant Mesoporous Ce‐doped Bioactive Glass Nanoparticles With Anti‐inflammatory and Pro‐osteogenic Activities,” Materials Today Bio 5 (2020): 100041, 10.1016/j.mtbio.2020.100041. PMC7083763 32211607

[30] F. Kurtuldu , N. Mutlu , R. P. Friedrich , et al., “Gallium‐containing Mesoporous Nanoparticles Influence in‐vitro Osteogenic and Osteoclastic Activity,” Biomaterials Advances 162 (2024): 213922, 10.1016/j.bioadv.2024.213922. 38878645

[31] R. A. Gittens , L. Scheideler , F. Rupp , et al., “A Review on the Wettability of Dental Implant Surfaces II: Biological and Clinical Aspects,” Acta Biomaterialia 10 (2014): 2907–2918, 10.1016/j.actbio.2014.03.032. 24709541 PMC4103435

[32] Y. Zhao , H. M. Wong , W. Wang , et al., “Cytocompatibility, Osseointegration, and Bioactivity of Three‐dimensional Porous and Nanostructured Network on Polyetheretherketone,” Biomaterials 34 (2013): 9264–9277, 10.1016/j.biomaterials.2013.08.071. 24041423

[33] L. E. Rustom , T. Boudou , S. Lou , et al., “Micropore‐induced Capillarity Enhances Bone Distribution *in Vivo* in Biphasic Calcium Phosphate Scaffolds,” Acta Biomaterialia 44 (2016): 144–154, 10.1016/j.actbio.2016.08.025. 27544807 PMC5045872

[34] J. Yan , W. Zhou , Z. Jia , et al., “Endowing Polyetheretherketone With Synergistic Bactericidal Effects and Improved Osteogenic Ability,” Acta Biomaterialia 79 (2018): 216–229, 10.1016/j.actbio.2018.08.037. 30172936

[35] S. Li , C. Jia , H. Han , Y. Yang , Y. Xiaowen , and Z. Chen , “Characterization and Biocompatibility of a Bilayer PEEK‐based Scaffold for Guiding Bone Regeneration,” BMC Oral Health 24 (2024): 1138, 10.1186/s12903-024-04909-z. 39334225 PMC11438270

[36] A. G. Al Lafi , “FTIR Spectroscopic Analysis of Ion Irradiated Poly (ether ether ketone),” Polymer Degradation and Stability 105 (2014): 122–133, 10.1016/j.polymdegradstab.2014.04.005.

[37] R. T. S. Muthu Lakshmi , V. Choudhary , and I. K. Varma , “Sulphonated Poly(ether ether ketone): Synthesis and Characterisation,” Journal of Materials Science 40 (2005): 629–636, 10.1007/s10853-005-6300-2.

[38] A. G. Al Lafi , “The sulfonation of poly(ether ether ketone) as investigated by two‐dimensional FTIR correlation spectroscopy,” Journal of Applied Polymer Science 132 (2015): 41242, 10.1002/app.41242.

[39] F. Baino and S. Yamaguchi , “The Use of Simulated Body Fluid (SBF) for Assessing Materials Bioactivity in the Context of Tissue Engineering: Review and Challenges,” Biomimetics 5 (2020): 57, 10.3390/biomimetics5040057. 33138246 PMC7709622

[40] T. Kokubo and H. Takadama , “How Useful Is SBF in Predicting in Vivo Bone Bioactivity?,” Biomaterials 27 (2006): 2907–2915, 10.1016/j.biomaterials.2006.01.017. 16448693

[41] R. J. Dekker , J. D. de Bruijn , M. Stigter , F. Barrere , P. Layrolle , and C. A. van Blitterswijk , “Bone Tissue Engineering on Amorphous Carbonated Apatite and Crystalline Octacalcium Phosphate‐coated Titanium Discs,” Biomaterials 26 (2005): 5231–5239, 10.1016/j.biomaterials.2005.01.057. 15792550

[42] Y. Deng , X. Shi , Y. Chen , et al., “Bacteria‐Triggered pH‐Responsive Osteopotentiating Coating on 3D‐Printed Polyetheretherketone Scaffolds for Infective Bone Defect Repair,” Industrial & Engineering Chemistry Research 59 (2020): 12123–12135, 10.1021/acs.iecr.0c02107.

[43] M. Edén , “Structure and Formation of Amorphous Calcium Phosphate and Its Role as Surface Layer of Nanocrystalline Apatite: Implications for Bone Mineralization,” Materialia 17 (2021): 101107, 10.1016/j.mtla.2021.101107.

[44] M. Gandolfi , P. Taddei , F. Siboni , et al., “Micro‐Topography and Reactivity of Implant Surfaces: An in Vitro Study in Simulated Body Fluid (SBF),” Microscopy and Microanalysis 21 (2015): 190–203, 10.1017/S1431927614014615. 25667970

[45] S. V. Dorozhkin , “Amorphous Calcium (ortho)Phosphates,” Acta Biomaterialia 6 (2010): 4457–4475, 10.1016/j.actbio.2010.06.031. 20609395

[46] H. Liu , X. Qu , H. Tan , et al., “Role of Polydopamine's Redox‐activity on Its Pro‐oxidant, Radical‐scavenging, and Antimicrobial Activities,” Acta Biomaterialia 88 (2019): 181–196, 10.1016/j.actbio.2019.02.032. 30818052

[47] F. Westhauser , S. Wilkesmann , Q. Nawaz , et al., “Effect of Manganese, Zinc, and Copper on the Biological and Osteogenic Properties of Mesoporous Bioactive Glass Nanoparticles,” Journal of Biomedical Materials Research Part A (2020): 1457–1467, 10.1002/jbm.a.37136. 33289275

[48] W. Xu , C. Xu , J. Yi , and H. Dai , “The Effect of Different Hydroxyapatite Microparticles on the Osteogenic Differentiation of MC3T3‐E1 Preosteoblasts,” Journal of Materials Chemistry B 6 (2018): 5234–5242, 10.1039/C8TB01352G. 32254760

[49] S. Jeon , J. H. Lee , H. J. Jang , et al., “Spontaneously Promoted Osteogenic Differentiation of MC3T3‐E1 Preosteoblasts on Ultrathin Layers of Black Phosphorus,” Materials Science and Engineering: C 128 (2021): 112309, 10.1016/j.msec.2021.112309. 34474860

[50] L. Yu , Y. Tian , Y. Qiao , and X. Liu , “Mn‐containing Titanium Surface With Favorable Osteogenic and Antimicrobial Functions Synthesized by PIII&D,” Colloids and Surfaces B: Biointerfaces 152 (2017): 376–384, 10.1016/j.colsurfb.2017.01.047. 28152461

[51] S. K. Ramasamy , A. P. Kusumbe , M. Schiller , et al., “Blood Flow Controls Bone Vascular Function and Osteogenesis,” Nature Communications 7 (2016): 13601, 10.1038/ncomms13601. PMC5150650 27922003

[52] C. Wang , J. Ying , X. Nie , et al., “Targeting Angiogenesis for Fracture Nonunion Treatment in Inflammatory Disease,” Bone Research 9 (2021): 29, 10.1038/s41413-021-00150-4. 34099632 PMC8184936

[53] S. Cao , H. Ma , and C. Wu , “Biomaterials‐modulated Multicellular Crosstalk in 3D Bioprinted Constructs,” Advanced Interventional Materials 1 (2026): 100008, 10.1016/j.advim.2025.100008.

[54] J. Wu , C. Qin , J. Ma , et al., “An Immunomodulatory Bioink With Hollow Manganese Silicate Nanospheres for Angiogenesis,” Applied Materials Today 23 (2021): 101015, 10.1016/j.apmt.2021.101015.

[55] H. Li and J. Chang , “Bioactive Silicate Materials Stimulate Angiogenesis in Fibroblast and Endothelial Cell co‐culture System Through Paracrine Effect,” Acta Biomaterialia 9 (2013): 6981–6991, 10.1016/j.actbio.2013.02.014. 23416471

[56] J. C. Bertels , G. He , and F. Long , “Metabolic Reprogramming in Skeletal Cell Differentiation,” Bone Research 12 (2024): 57, 10.1038/s41413-024-00374-0. 39394187 PMC11470040

[57] W.‐C. Lee , A. R. Guntur , F. Long , and C. J. Rosen , “Energy Metabolism of the Osteoblast: Implications for Osteoporosis,” Endocrine Reviews 38 (2017): 255–266, 10.1210/er.2017-00064. 28472361 PMC5460680

[58] J. R. Whiteford , V. Behrends , H. Kirby , M. Kusche‐Gullberg , T. Muramatsu , and J. R. Couchman , “Syndecans Promote Integrin‐mediated Adhesion of Mesenchymal Cells in Two Distinct Pathways,” Experimental Cell Research 313 (2007): 3902–3913, 10.1016/j.yexcr.2007.08.002. 17870067

[59] L. Yang , H. Chen , C. Yang , et al., “Research progress on the regulatory mechanism of integrin‐mediated mechanical stress in cells involved in bone metabolism,” Journal of Cellular and Molecular Medicine 28 (2024): 18183, 10.1111/jcmm.18183. PMC10951882 38506078

[60] A. Bozec , L. Bakiri , M. Jimenez , T. Schinke , M. Amling , and E. F. Wagner , “Fra‐2/AP‐1 Controls Bone Formation by Regulating Osteoblast Differentiation and Collagen Production,” Journal of Cell Biology 190 (2010): 1093–1106, 10.1083/jcb.201002111. 20837772 PMC3101588

[61] H. Iwakawa and Y. Tomari , “Life of RISC: Formation, Action, and Degradation of RNA‐induced Silencing Complex,” Molecular Cell 82 (2022): 30–43, 10.1016/j.molcel.2021.11.026. 34942118

[62] J. B. Lian , G. S. Stein , A. J. van Wijnen , et al., “MicroRNA Control of Bone Formation and Homeostasis,” Nature Reviews Endocrinology 8 (2012): 212–227, 10.1038/nrendo.2011.234. PMC3589914 22290358

[63] Z. Zheng , Y. He , L. Long , et al., “Involvement of PI3K/Akt Signaling Pathway in Promoting Osteogenesis on Titanium Implant Surfaces Modified With Novel Non‐thermal Atmospheric Plasma,” Frontiers in Bioengineering and Biotechnology 10 (2022): 975840, 10.3389/fbioe.2022.975840. 36185461 PMC9523010

[64] Z. Saidak , C. L. Henaff , S. Azzi , et al., “Wnt/β‐Catenin Signaling Mediates Osteoblast Differentiation Triggered by Peptide‐induced α5β1 Integrin Priming in Mesenchymal Skeletal Cells,” Journal of Biological Chemistry 290 (2015): 6903–6912, 10.1074/jbc.M114.621219. 25631051 PMC4358115

[65] M. Li , R. Guo , J. Wei , et al., “Polydopamine‐based Nanoplatform for Photothermal Ablation With Long‐term Immune Activation Against Melanoma and Its Recurrence,” Acta Biomaterialia 136 (2021): 546–557, 10.1016/j.actbio.2021.09.014. 34536603

[66] J. Lu , L. Cai , Y. Dai , et al., “Polydopamine‐Based Nanoparticles for Photothermal Therapy/Chemotherapy and their Synergistic Therapy With Autophagy Inhibitor to Promote Antitumor Treatment,” The Chemical Record 21 (2021): 781–796, 10.1002/tcr.202000170. 33634962

[67] Z. Chen , Z. Li , C. Li , et al., “Manganese‐containing Polydopamine Nanoparticles as Theranostic Agents for Magnetic Resonance Imaging and Photothermal/Chemodynamic Combined Ferroptosis Therapy Treating Gastric Cancer,” Drug Delivery 29 (2022): 1201–1211, 10.1080/10717544.2022.2059124. 35403518 PMC9004524

[68] J. Xi , L. Da , C. Yang , et al., “Mn^2+^‐coordinated PDA@DOX/PLGA nanoparticles as a smart theranostic agent for synergistic chemo‐photothermal tumor therapy,” International Journal of Nanomedicine 12 (2017): 3331–3345, 10.2147/IJN.S132270. 28479854 PMC5411169

[69] H. Karkhanechi , R. Takagi , and H. Matsuyama , “Biofouling Resistance of Reverse Osmosis Membrane Modified With Polydopamine,” Desalination 336 (2014): 87–96, 10.1016/j.desal.2013.12.033.

[70] S. Alfei , G. C. Schito , A. M. Schito , and G. Zuccari , “Reactive Oxygen Species (ROS)‐Mediated Antibacterial Oxidative Therapies: Available Methods to Generate ROS and a Novel Option Proposal,” International Journal of Molecular Sciences 25 (2024): 7182, 10.3390/ijms25137182. 39000290 PMC11241369

[71] F. Kurtuldu , N. Mutlu , M. Michálek , et al., “Cerium and Gallium Containing Mesoporous Bioactive Glass Nanoparticles for Bone Regeneration: Bioactivity, Biocompatibility and Antibacterial Activity,” Materials Science and Engineering: C 124 (2021): 112050, 10.1016/j.msec.2021.112050. 33947544

[72] T. Du , S. Zhao , W. Dong , et al., “Surface Modification of Carbon Fiber‐Reinforced Polyetheretherketone With MXene Nanosheets for Enhanced Photothermal Antibacterial Activity and Osteogenicity,” ACS Biomaterials Science & Engineering 8 (2022): 2375–2389, 10.1021/acsbiomaterials.2c00095. 35652599

[73] S. Kandasamy , V. Narayanan , and S. Sumathi , “Zinc and Manganese Substituted Hydroxyapatite/CMC/PVP Electrospun Composite for Bone Repair Applications,” International Journal of Biological Macromolecules 145 (2020): 1018–1030, 10.1016/j.ijbiomac.2019.09.193. 31726129

[74] C.‐F. Tseng , Y.‐C. Fei , and Y.‐J. Chou , “Investigation of in Vitro Bioactivity and Antibacterial Activity of Manganese‐doped Spray Pyrolyzed Bioactive Glasses,” Journal of Non‐Crystalline Solids 549 (2020): 120336, 10.1016/j.jnoncrysol.2020.120336.

[75] D. Marovic , M. Par , T. T. Tauböck , et al., “Impact of Copper‐Doped Mesoporous Bioactive Glass Nanospheres on the Polymerisation Kinetics and Shrinkage Stress of Dental Resin Composites,” International Journal of Molecular Sciences 23 (2022): 8195, 10.3390/ijms23158195. 35897771 PMC9332616

[76] R. D'Amato , G. Quaglia , R. Selvaggi , F. Marmottini , and L. Latterini , “Role of Surface Defects on Photoinduced Reactivity in SiO_2_ Nanoparticles,” Inorganics 11 (2023): 430, 10.3390/inorganics11110430.

[77] J. Liebscher , R. Mrówczyński , H. A. Scheidt , et al., “Structure of Polydopamine: A Never‐Ending Story?,” Langmuir 29 (2013): 10539–10548, 10.1021/la4020288. 23875692

[78] X. Ding , Y. Zhang , J. Ling , and C. Lin , “Rapid Mussel‐inspired Synthesis of PDA‐Zn‐Ag Nanofilms on TiO_2_ Nanotubes for Optimizing the Antibacterial Activity and Biocompatibility by Doping Polydopamine With Zinc at a Higher Temperature,” Colloids and Surfaces B: Biointerfaces 171 (2018): 101–109, 10.1016/j.colsurfb.2018.07.014. 30015139

[79] H. A. Lee , E. Park , and H. Lee , “Polydopamine and Its Derivative Surface Chemistry in Material Science: A Focused Review for Studies at KAIST,” Advanced Materials 32 (2020): 1907505, 10.1002/adma.201907505. 32134525

[80] M. Battaglini , M. Emanet , A. Carmignani , and G. Ciofani , “Polydopamine‐based Nanostructures: A New Generation of Versatile, Multi‐tasking, and Smart Theranostic Tools,” Nano Today 55 (2024): 102151, 10.1016/j.nantod.2024.102151.

[81] Y. Zou , P. Yang , L. Yang , et al., “Boosting Solar Steam Generation by Photothermal Enhanced Polydopamine/Wood Composites,” Polymer 217 (2021): 123464, 10.1016/j.polymer.2021.123464.

[82] Y. Zou , X. Chen , P. Yang , et al., “Regulating the Absorption Spectrum of Polydopamine,” Science Advances 6 (2020): abb4696, 10.1126/sciadv.abb4696. PMC7473670 32917617

[83] C. Daniel , “Absorption Spectroscopy, Emissive Properties, and Ultrafast Intersystem Crossing Processes in Transition Metal Complexes: TD‐DFT and Spin‐Orbit Coupling,” in Density‐Functional Methods for Excited States, ed. N. Ferré , M. Filatov , and M. Huix‐Rotllant , (Springer International Publishing, 2016), 377–413, 10.1007/128_2015_635. 26129697

[84] H. Ma , S. Long , J. Cao , et al., “New Cy5 photosensitizers for cancer phototherapy: A low singlet–triplet gap provides high quantum yield of singlet oxygen,” Chemical Science 12 (2021): 13809–13816, 10.1039/d1sc04570a. 34760166 PMC8549779

[85] Q. Wu , L. Tan , X. Liu , et al., “The Enhanced near‐infrared Photocatalytic and Photothermal Effects of MXene‐based Heterojunction for Rapid Bacteria‐killing,” Applied Catalysis B: Environmental 297 (2021): 120500, 10.1016/j.apcatb.2021.120500.

[86] X. Cui , Q. Ruan , X. Zhuo , et al., “Photothermal Nanomaterials: A Powerful Light‐to‐Heat Converter,” Chemical Reviews 123 (2023): 6891–6952, 10.1021/acs.chemrev.3c00159. 37133878 PMC10273250

[87] S. Banerjee , S. C. Pillai , P. Falaras , K. E. O'Shea , J. A. Byrne , and D. D. Dionysiou , “New Insights Into the Mechanism of Visible Light Photocatalysis,” The Journal of Physical Chemistry Letters 5 (2014): 2543–2554, 10.1021/jz501030x. 26277942

[88] W. Yang , H. Jia , T. Li , Y. Liu , and Y. Li , “Distinct Pathways for Superoxide Radical Generation Induced by Mn and Cu‐based Catalysts in Electro‐Fenton Like Process,” Journal of Environmental Management 378 (2025): 124664, 10.1016/j.jenvman.2025.124664. 40031417

[89] L.‐F. Xie , L. Niu , T.‐T. Xie , et al., “Regulating the Combinations of Donor and Acceptor Units via DFT Calculations for Photocatalysts With Efficient Electron–Hole Separation and Transfer Dynamics,” ACS Applied Materials & Interfaces 17 (2025): 22646–22656, 10.1021/acsami.5c00291. 40179347

[90] X. Hu , Z. Fang , F. Sun , et al., “Deciphering Oxygen‐Independent Augmented Photodynamic Oncotherapy by Facilitating the Separation of Electron‐Hole Pairs,” Angewandte Chemie International Edition 63 (2024): 202401036, 10.1002/anie.202401036. 38362791

[91] L. Huang , Y. Xuan , Y. Koide , T. Zhiyentayev , M. Tanaka , and M. R. Hamblin , “Type I and Type II mechanisms of antimicrobial photodynamic therapy: An in vitro study on gram‐negative and gram‐positive bacteria,” Lasers in Surgery and Medicine 44 (2012): 490–499, 10.1002/lsm.22045. 22760848 PMC3428129

[92] C. He , P. Feng , M. Hao , et al., “Nanomaterials in Antibacterial Photodynamic Therapy and Antibacterial Sonodynamic Therapy,” Advanced Functional Materials 34 (2024): 2402588, 10.1002/adfm.202402588.

[93] P. Wang , C. Liang , J. Zhu , et al., “Manganese‐Based Nanoplatform as Metal Ion‐Enhanced ROS Generator for Combined Chemodynamic/Photodynamic Therapy,” ACS Applied Materials & Interfaces 11 (2019): 41140–41147, 10.1021/acsami.9b16617. 31603650

[94] X. Yang , Q. Wang , Y. Zhang , et al., “A Dual‐functional PEEK Implant Coating for Anti‐bacterial and Accelerated Osseointegration,” Colloids and Surfaces B: Biointerfaces 224 (2023): 113196, 10.1016/j.colsurfb.2023.113196. 36764204

[95] L. Wang , H. He , X. Yang , et al., “Bimetallic Ions Regulated PEEK of Bone Implantation for Antibacterial and Osteogenic Activities,” Materials Today Advances 12 (2021): 100162, 10.1016/j.mtadv.2021.100162.

[96] A. D. Laurent and D. Jacquemin , “TD‐DFT Benchmarks: A Review,” International Journal of Quantum Chemistry 113 (2013): 2019–2039, 10.1002/qua.24438.

