# Supplementary material for: An Ion‐Based Strategy Toward Synergistic Surface Functionalization Combining the Osteogenic Properties and NIR‐Mediated Antibacterial Activity of PEEK
Source: Adv Mater. 2026 Jul 9;38(45):e73945. doi: 10.1002/adma.73945 (PMC13472038; doi:10.1002/adma.73945)
Supplement: Supplementary file 1 [file ADMA-38-e73945-s001.docx]

Supporting Information

An ion-based strategy toward synergistic surface functionalization combining the osteogenic properties and NIR-mediated antibacterial activity of PEEK

Zhiyan Xu, Ondřej Adam, Marek Doubrava, Ana M. Beltrán, Ivo Dlouhý, Xin Liu*, Baiyan Sui*, and Aldo R. Boccaccini*

**Figure S1.** **a)** The enlarged and overlapping views of the FTIR spectra on the same baseline (from 1400 to 400 cm^-1^) highlight the bands at approximately 1109, 1095, and 1028 cm^-1^ and the absorbance variations of bands at around 1051 cm^-1^ and 443 cm^-1^ among the samples; **b)** The bands' variation is highlighted at approximately 3300 cm^-1^.

**Figure S2.** High-resolution **a)** Si 2p and **b)** Ca 2p XPS spectra of Mn@p/MBG-SPEEK.

**Figure S3.** EDS spectra of p-SPEEK, p/MBG-SPEEK, and Mn@p/MBG-SPEEK after immersion in SBF for 7 days. The Ca/P ratio of the Ca-P clusters is calculated and presented, respectively.

**Figure S4.** Cell viability of MC3T3-E1 co-cultured with PEEK samples for 1 and 3 days (*n* = 3). Data are presented as mean ± SD. Statistical analysis was performed using two-way ANOVA followed by Tukey’s HSD post hoc test. ns: *p* > 0.05, ***p* < 0.01, and ****p* < 0.001; ###*p* < 0.001, day 1 *vs.* day 3.

**Figure S5.** Evaluation of biofunctional component release profiles and surface stability of PEEK-based samples after immersion in cell culture medium up to 7 days. **a)** Cumulative release profiles of Si, Ca, and Mn (*n* = 3, data are presented as mean ± SD); **b)** XPS survey spectra, **c)** high-resolution Mn 2p XPS spectrum and **d)** high-resolution N 1s XPS spectra of Mn@p/MBG-SPEEK; **e)** SEM images of PEEK, p-SPEEK, p/MBG-SPEEK, and Mn@p/MBG-SPEEK surfaces after immersion; **f)** EDS spectrum and elemental mappings of p/MBG-SPEEK surface after immersion. Clear N signals were still observed in the PDA-containing samples after immersion, and Mn 2p signals remained detectable on Mn@p/MBG-SPEEK, indicating retention of PDA and/or Mn-chelated PDA components on the surface. SEM-EDS analysis further showed no obvious coating collapse or massive loss of MBGNs-related surface features after immersion.

**Figure S6.** Endothelial cytocompatibility and quantitative analysis of tube formation. **a)** Cell viability of HUVECs cultured on different PEEK samples for 1 and 3 days. **b-d)** Quantitative analysis of **b)** number of junctions, **c)** number of meshes, and **d)** relative tube length from HUVECs-GFP tube formation after treatment with extracts from different PEEK-based samples. Data are presented as mean ± SD (*n* = 3). Statistical significance was evaluated by one-way ANOVA followed by Tukey’s HSD post hoc test. **p* < 0.05, ***p* < 0.01, and ****p* < 0.001 *vs*. PEEK; #*p* < 0.05, and ###*p* < 0.001 between indicated groups.

**Figure S7.** Transcriptomic profiling and GO analysis of MC3T3-E1 cells cultured on different PEEK-based substrates. **a)** Hierarchical clustering heatmap of DEGs illustrating distinct transcriptional signatures among groups. **b)** GO Level 2 classification of DEGs into biological process (BP), molecular function (MF), and cellular component (CC) categories.

**Figure S8.** KEGG pathway enrichment analysis of upregulated DEGs in MC3T3-E1 cells cultured on different PEEK samples. **a)** Classified KEGG pathway enrichment for PEEK *vs.* p-SPEEK, and **b)** corresponding Sankey diagram illustrating the mapping of enriched pathways to functional categories; **c)** Classified KEGG pathway enrichment for PEEK *vs.* p/MBG-SPEEK, and **d)** corresponding Sankey diagram illustrating the mapping of enriched pathways to functional categories; **e)** Classified KEGG pathway enrichment for PEEK *vs.* Mn@p/MBG-SPEEK, and **f)** corresponding Sankey diagram illustrating the mapping of enriched pathways to functional categories.

**Figure S9.** The representative real-time infrared thermal images of PEEK samples with NIR laser at 1.0 W cm^-2^ under dry conditions at different time intervals.

**Figure S10.** ESR spectra of PEEK-based samples recorded under **a)** hydroxyl radical- and **b)** singlet oxygen-probing conditions without and with 808 nm NIR irradiation for 5 min.

**Figure S11**. Constructed chemical structures of SPEEK-C, p-SPEEK-C, and Mn@p-SPEEK-C. The negative Gibbs free energy values for the stepwise transformations (from SPEEK-C to p-SPEEK-C and then to Mn@p-SPEEK-C) with increasingly larger magnitudes suggest that each process occurs spontaneously and that the stability of the system increases along the transformation pathway. Accordingly, Mn@p-SPEEK-C presents the most thermodynamically stable configuration among the three structures.

**Figure S12.** Mulliken spin population analysis of the stable wavefunction of Mn@p-SPEEK-C and charge analysis of Mn atoms. After Mn incorporation, the ground state adopts a symmetry-broken singlet configuration. Each Mn center contributes one unpaired electron, and the four unpaired electrons pair antiferromagnetically in opposite spin directions, resulting in an overall singlet system.

**Figure S13.** The electron (green isosurface)-hole (blue isosurface) density difference plots for sequentially increasing singlet excited states from S1 to S12 in p-SPEEK-C. The electron-hole distribution analyses demonstrate the separation and transfer processes of electrons and holes in different excited states.

**Figure S14.** The electron (green isosurface)-hole (blue isosurface) density difference plots for sequentially increasing quintet excited states from Q1 to Q10 in Mn@p-SPEEK-C. The electron-hole distribution analyses demonstrate the separation and transfer processes of electrons and holes in different excited states.

**Table S1.** Chemical composition estimated from EDS data and determined from ICP-OES results, BET specific surface area (SSA), and pore volume of MBGNs.

| **Designation** |  | **SiO_2_**  **[mol%]** | **CaO**  **[mol%]** | **SSA**  **[m^2^ g^-1^]** | **Pore volume**  **[cm^3^ g^-1^]** |
| --- | --- | --- | --- | --- | --- |
| MBGNs | EDS | 90.4 ± 0.3 | 9.6 ± 0.3 | 326 ± 4 | 0.36 |
|  | ICP-OES | 91.1 | 8.9 |  |  |

**Table S2.** Summary of the excitation energies, oscillator strengths, and dominant orbital contributions in p-SPEEK-C obtained by TD-DFT calculations (HOMO is denoted as H; LUMO is denoted as L).

| **Electronic transition** | **Excitation energy [eV]** | **Excitation energy [nm]** | **Oscillator strength** | **Dominant orbital contributions** |
| --- | --- | --- | --- | --- |
| S0 → S1 | 3.5046 | 353.78 | 0.0083 | H-3 → L 99.0% |
| S0 → S2 | 3.5741 | 346.90 | 0.0040 | H → L+6 95.6% |
| S0 → S3 | 3.6025 | 344.16 | 0.0042 | H-2 → L+2 85.1%, H-2 → L+4 9.1%, H-2 → L+3 5.1% |
| S0 → S4 | 3.6063 | 343.80 | 0.0040 | H-1 → L+3 52.9%, H-1 → L+4 34.3%, H-1 → L+5 12.4% |
| S0 → S5 | 3.7271 | 332.66 | 0.0075 | H → L+7 85.1%, H → L+5 5.6% |
| S0 → S6 | 3.7301 | 332.39 | 0.0081 | H-1 → L+5 69.2%, H-1 → L+3 19.6% |
| S0 → S7 | 3.7311 | 332.30 | 0.0055 | H-2 → L+4 49.0%, H-2 → L+3 20.7%, H-2 → L+2 10.5%, H-2 → L+1 8.1% |
| S0 → S8 | 3.7450 | 331.07 | 0.0050 | H-3 → L+1 89.6% |
| S0 → S9 | 3.7834 | 327.71 | 0.0032 | H-17 → L+7 36.3%, H-9 → L+7 6.7%, H-4 → L+7 5.5%, H-5 → L+7 5.3%, H-7 → L+7 5.1% |
| S0 → S10 | 3.7841 | 327.65 | 0.0038 | H-18 → L+5 16.7%, H-18 → L+3 10.9%, H-17 → L+7 6.1%, H-19 → L+4 5.9% |
| S0 → S11 | 3.7845 | 327.61 | 0.0070 | H-21 → L+1 25.3%, H-11 → L+1 8.1%, H-18 → L+5 7.8% |
| S0 → S12 | 3.7851 | 327.56 | 0.0031 | H-21 → L+1 17.6%, H-19 → L+4 12.5%, H-19 → L+2 8.1%, H-19 → L+3 6.7%, H-11 → L+1 5.7% |
| S0 → T1 | 2.9762 | 416.59 | 0.0000 | H-4 → L+7 13.5%, H-5 → L+7 13.0%, H-7 → L+7 7.1%, H-17 → L+7 6.6%, H-9 → L+7 6.2% |
| S0 → T2 | 2.9769 | 416.49 | 0.0000 | H-6 → L+3 9.1%, H-8 → L+4 6.2%, H-6 → L+5 5.5% |
| S0 → T3 | 2.9776 | 416.39 | 0.0000 | H-6 → L+5 10.6%, H-8 → L+2 6.7%, H-8 → L+3 6.4%, H-8 → L+4 5.4% |
| S0 → T4 | 2.9805 | 415.98 | 0.0000 | H-11 → L+1 35.2%, H-21 → L+1 9.7%, H-14 → L+1 6.3% |
| S0 → T5 | 3.2511 | 381.36 | 0.0000 | H-5 → L+6 9.4% |

**Table S3.** Summary of the excitation energies, oscillator strengths, and dominant orbital contributions in Mn@p-SPEEK-C obtained by TD-DFT calculations (alpha-HOMO is denoted as Ha; alpha-LUMO is denoted as La; beta-HOMO is denoted as Hb; beta-LUMO is denoted as Lb).

| **Electronic transition** | **Excitation energy [eV]** | **Excitation energy [nm]** | **Oscillator strength** | **Dominant orbital contributions** |
| --- | --- | --- | --- | --- |
| S0 → Q1 | 0.0305 | 40650.56 | 0.0000 | Hb → Lb 100.0% |
| S0 → Q2 | 0.1894 | 6546.16 | 0.0000 | Hb-1 → Lb 100.0% |
| S0 → Q3 | 0.2383 | 5202.86 | 0.0000 | Hb-2 → Lb 100.0% |
| S0 → Q4 | 0.4653 | 2664.61 | 0.0000 | Hb → Lb+1 100.0% |
| S0 → Q5 | 0.5224 | 2373.36 | 0.0000 | Hb-3 → Lb 90.7%, Hb-4 → Lb 9.3% |
| S0 → Q6 | 0.5294 | 2341.98 | 0.0000 | Ha → La 100.0% |
| S0 → Q7 | 0.6002 | 2065.71 | 0.0000 | Hb-4 → Lb 90.7%, Hb-3 → Lb 9.3% |
| S0 → Q8 | 0.6262 | 1979.95 | 0.0000 | Hb-1 → Lb+1 100.0% |
| S0 → Q9 | 0.6733 | 1841.44 | 0.0000 | Hb-2 → Lb+1 100.0% |
| S0 → Q10 | 0.8220 | 1509.73 | 0.0000 | Hb-5 → Lb 100.0% |

**Table S4.** The sequences for MC3T3-E1 cells PCR primers.

| Gene |  | Primer sequence |
| --- | --- | --- |
| ALP | Forward | 5’-CCAACTCTTTTGTGCCAGAGA-3’ |
|  | Reverse | 5’-GGCTACATTGGTGTTGAGCTTTT-3’ |
| OCN | Forward | 5’-CTGACCTCACAGATCCCAAGC-3’ |
|  | Reverse | 5’-TGGTCTGATAGCTCGTCACAAG-3’ |
| OPN | Forward | 5’-AGCAAGAAACTCTTCCAAGCAA-3’ |
|  | Reverse | 5’-GTGAGATTCGTCAGATTCATCCG-3’ |
| Runx2 | Forward | 5’-ATGCTTCATTCGCCTCACAAA-3’ |
|  | Reverse | 5’-GCACTCACTGACTCGGTTGG-3’ |
| GAPDH | Forward | 5’-AGGTCGGTGTGAACGGATTTG-3’ |
|  | Reverse | 5’-TGTAGACCATGTAGTTGAGGTCA-3’ |

**Table S5.** The sequences for HUVECs PCR primers.

| Gene |  | Primer sequence |
| --- | --- | --- |
| VEGF | Forward | 5’- AGGGCAGAATCATCACGAAGT-3’ |
|  | Reverse | 5’- AGGGTCTCGATTGGATGGCA-3’ |
| CD31 | Forward | 5’- AACAGTGTTGACATGAAGAGCC-3’ |
|  | Reverse | 5’- TGTAAAACAGCACGTCATCCTT-3’ |
| GAPDH | Forward | 5’- GGAGCGAGATCCCTCCAAAAT-3’ |
|  | Reverse | 5’- GGCTGTTGTCATACTTCTCATGG-3’ |
